# Supplementary material for: Tetrasubstituted Pyrrole Derivative Mimetics of Protein–Protein Interaction Hot-Spot Residues: A Promising Class of Anticancer Agents Targeting Melanoma Cells
Source: Molecules. 2023 May 18;28(10):4161. doi: 10.3390/molecules28104161 (PMC10223356; doi:10.3390/molecules28104161)
Supplement: Supplementary file 1 [file molecules-28-04161-s001.zip › molecules-2343793-supplementary.pdf]

Supporting Information

Tetrasubstituted pyrrole derivatives mimetics of protein-protein interaction hot spot residues: a promising class of anticancer agents targeting melanoma cells

Marco Persico [1#], Paola Galatello [2#], Maria Grazia Ferraro [1], Carlo Irace [1], Avazbek Abduvakhidov [1], Oleh Tkachuk [1], Maria Luisa d'Aulisio Garigliota [2], Marialuisa Piccolo [1], Pietro Campiglia [2], Patrizia Iannece [3], Varra Michela [1]*, Anna Ramunno [2]* and Caterina Fattorusso [1]*

TABLE OF CONTENT

Figure S1	Graphical representation of the absorption of PBS measured at 640 vs µL of added DMSO solutions	2-3
Figure S2	Antiproliferative activity during bioscreen in vitro	4
Figure S3	Comparison of the conformational enantiomers of the global minimum conformers of compound **10a**	5
Figure S4	Conformers of TSP conformational families of 10a, 9g and 10b	6
Figure S5	Superimposition between the PM6 and the DFT TCC and TCT lowest energy conformer of **1**	7
Figure S6	Sequence alignment between the human $p14^{ARF}$ and the mouse $p19^{ARF}$ tumor suppressor protein	8
Tables S1-S12	ΔE_{GM} values (kcal/mol), torsional angle values (degrees) and occurrence rates (%) of conformational families considering MM conformers within 5 kcal/mol from the global minimum	9-20
Tables S13-S23	Conformational energy, structural classification, and intramolecular distances (Å) of calculated DFT conformers	21-31
Table S24	TSP conformers grouped according to the intramolecular distances between the X, Y, and Z rings.	32
Table S25	Distances between the centroids of the side chains of the hot-spot residues of the considered PPI motifs.	33
Table S26	Calculated RMSD (Å) by fitting the centroids of the aromatic rings X, Y, and Z of the selected DFT conformers on the centroids of the side chains of the considered PPI motif	34
Table S27	Calculated RMSD (Å) by fitting the centroids of the aromatic rings X, Y, and Z of the selected DFT conformers on the centroids of the side chains of the considered PPI motif on human $p14^{ARF}$	35
Table S28	$cLogD_{7.4}$ of compounds **1**, **9a-i,** and **10a-b**	36
Scheme S1	Synthesis of diketone derivatives **5b,c**	37
	NMR Spectra of **9a-i**, **10a**, **±10b**	38-48

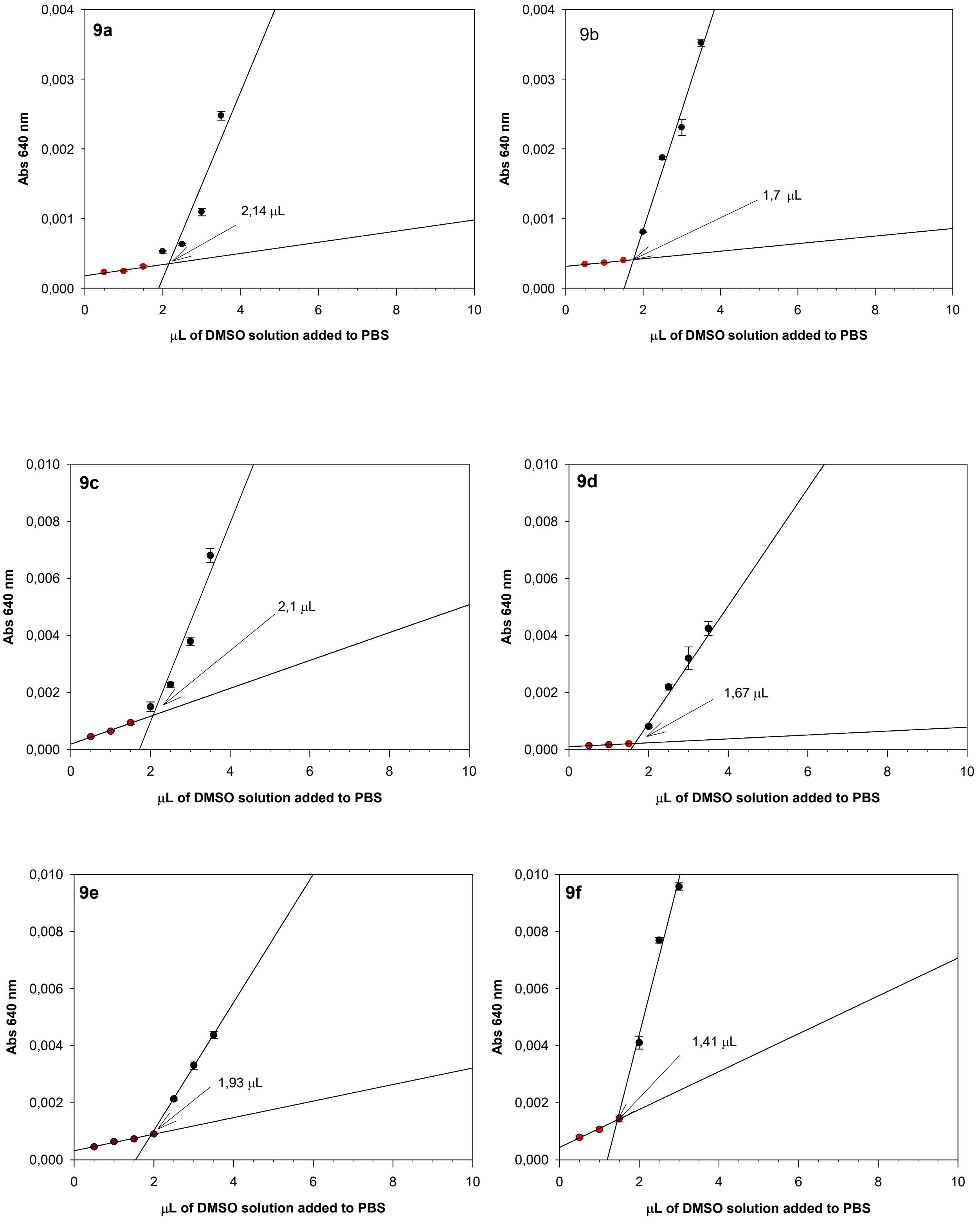
9a
2,14 μL
9b
1,7 μL
9c
2,1 μL
9d
1,67 μL
9e
1,93 μL
9f
1,41 μL
Abs 640 nm
μL of DMSO solution added to PBS

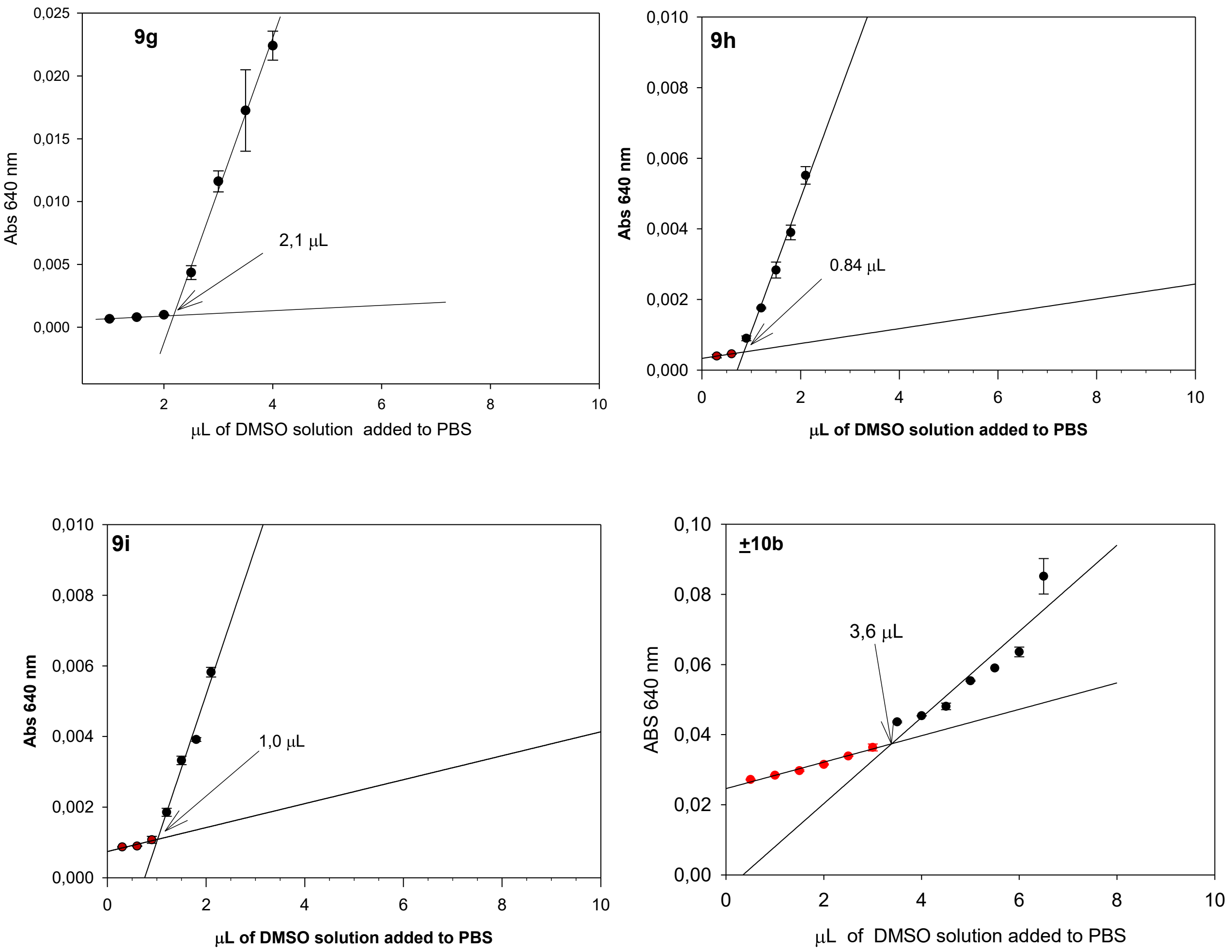

Figure S1. Graphical representation of the absorption of PBS measured at 640 vs μL of added DMSO solutions containing the species indicated in each panel. DMSO solutions (2,6 mM, for **9a**, **9b**, **9c**, **9d**, **9e**, **9f**, **±10b** or 1,0 mM for **9g**, **9h**, **9i**) were added to 1400 μL of PBS 0.5 at time. In all cases the final % (Vol/Vol) of DMSO in PBS was ≤0.6%

(A)

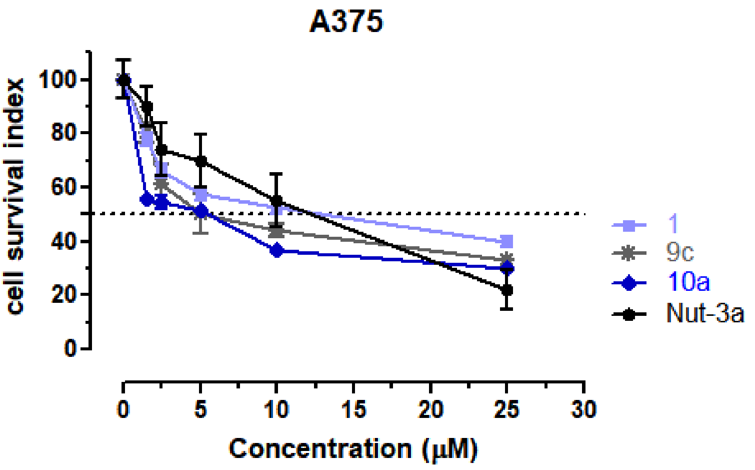

(B)

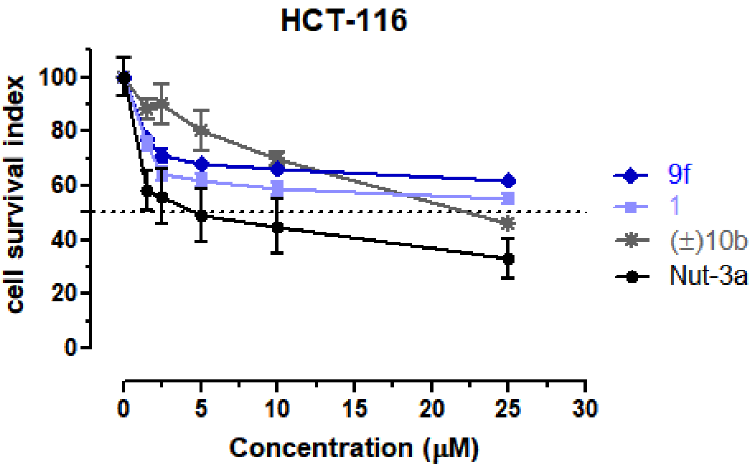

Figure S2. Antiproliferative activity during bioscreen *in vitro*. Cell survival index, evaluated by the MTT assay and the estimation of live/dead cell ratio, following 48 h of incubation with the indicated concentrations (the range 1→25 µM has been explored) of compounds **1**, **9c**, and **10a** for A375 cells **(A)**, and of compounds **9f**, **1** and **(±)10b** for HCT-116 cells **(B)**, respectively, as indicated in the legends. Nutlin-3a (**Nut-3a**) was used as positive control for cytotoxic and antiproliferative effects. Data are expressed as percentage of untreated control cells and are reported as mean of five independent experiments ± SEM (n = 30).

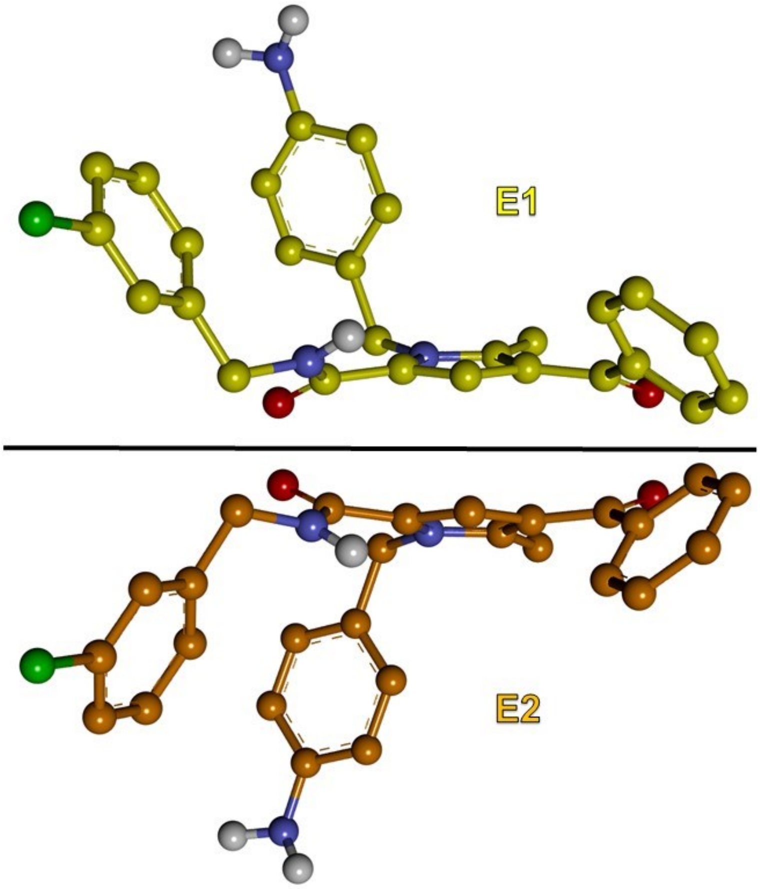

Figure S3. Comparison of the conformational enantiomers of the global minimum conformer of compound **10a.** Structures are displayed as ball and sticks. Heteroatoms are colored by atom type: O, red; N, blue and Cl, light green. Hydrogens are omitted for clarity with the exception of those bonded to the amide and amine nitrogen.

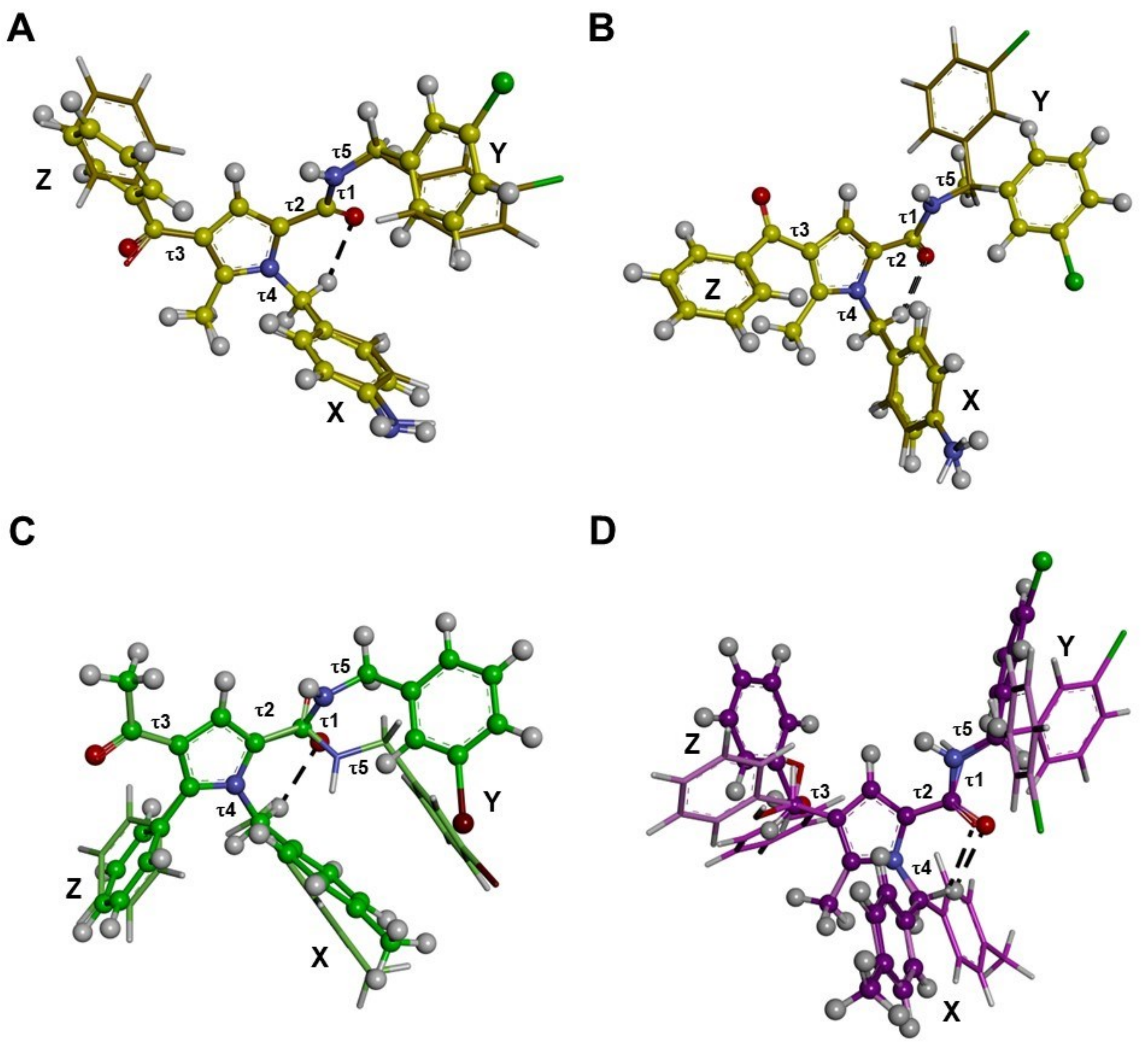

Figure S4. TSP conformers: TCC I (yellow) and TCC II (dark yellow) (**10a**; A); TCT I (yellow) and TCT III (dark yellow) (**10a**; B); TCC I (green) and TTC IV (light green) (**9g**; C); TCG$^-$ (violet), TCA$^-$ (pink) and TCG$^+$ (magenta) (**R-10b**; D). The lowest energy conformers of the family are displayed in ball&stick and the others (if any) in stick. The τ1-τ5 torsion angles and the pharmacophoric rings are labelled. Heteroatoms are colored by atom type: O, red; N, blue; Cl, green; Br, brown. The intra-molecular hydrogen-bond interactions are evidenced with a black dashed line.

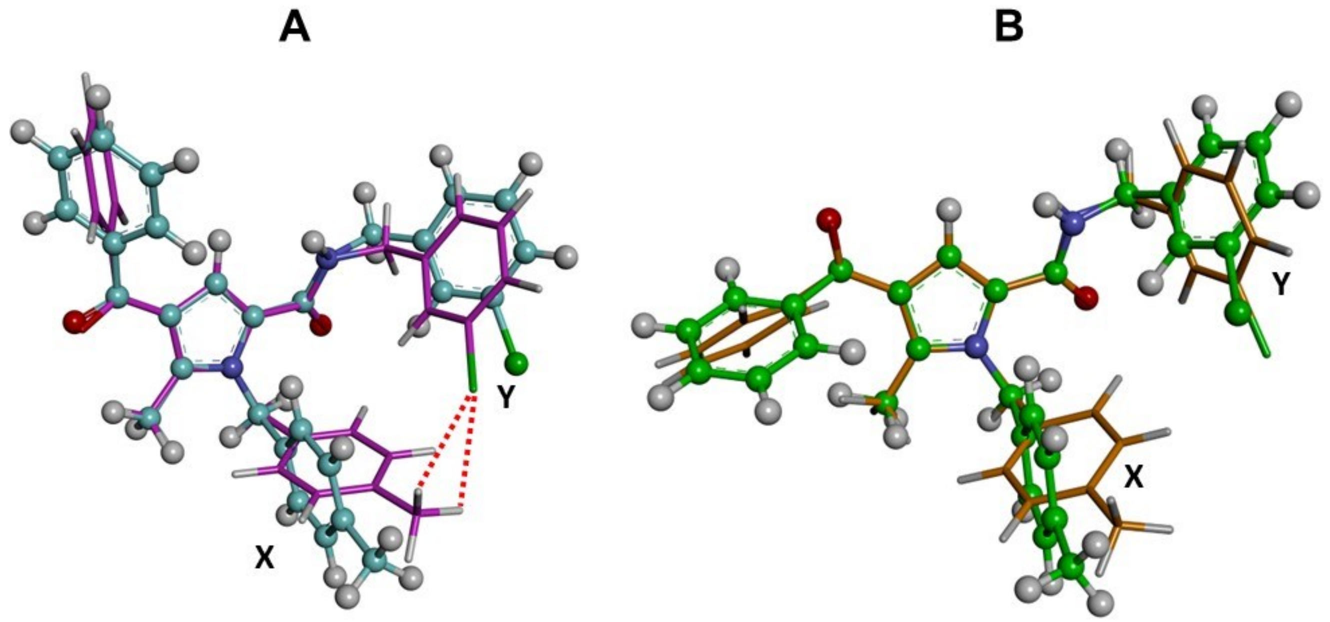

Figure S5. A: Comparison between the PM6 (stick and carbon atoms in magenta) and the DFT (ball&stick and carbon atoms in light blue) TCC I conformer of **1**. B: Comparison between the PM6 (stick and carbon atoms in orange) and the DFT (ball&stick and carbon atoms in green) TCT I conformer of **1**. Heteroatoms are colored by atom type: O, red; N, blue; Cl, light green. The intra-molecular hydrogen-bond interactions between methyl group and chlorine atom are evidenced with red dashed lines.

```
                 α-helix H1    α-helix H2
p19ARF_mouse     1   MGRRFLVTVRIQRAGRPLQERVFLVKFVRSRRPRTASCALAFVNMLLRLERILRRGPHRNP-GPGDDDGQ   69
p14ARF_human     1   MVRRFLVTLRIRRACGPPRVRVFVVHIPRLTGEWAAPGAPAAVALVLMLLRSQRLGQQPLPRRPGHDDGQ   70
Consensus aa:        MsRRFLVTlRIpRAt.P.p.RVFlV+hsR.p...hAstA.AhVshlL.LbR.bR.G.p..P..PGcDDGQ

p19ARF_mouse    70   RSRSSSSAQLRCRFELRGPHYLLPPGARRSAGRLPGHAGGAARVRGSAGCARCLGSPAARLGPRAGTSRH  139
p14ARF_human    71   RPSGGAAAAPRRGAQLRRPRHSHPTRARRCPGGLPGHAGGAAPGRGAAGRARCLGPSARGPG--------  132
Consensus aa:        RsptttA..R..hpLR.P+@.hPs.ARRtsG.LPGHAGGAA.sRGtAG.ARCLGssA...G........

p19ARF_mouse   140   RAIFAFRWVLFVFRWVVFVYRWERRPDRRA  169
p14ARF_human         ------------------------------
Consensus aa:        ..............................
```

Figure S6. Sequence alignment of the human p14ARF and the mouse p19ARF tumor suppressor protein. The first two N-terminal helices of p14ARF (H1 (4-15) and H2 (20-30)) involved in the interaction with MDM2 are evidenced with a red box.

Table S1. ΔE_{GM} values (kcal/mol), torsional angle values (degrees) and occurrence rates (%) of conformational families of **1** considering MM conformers within 5 kcal/mol from the global minimum.

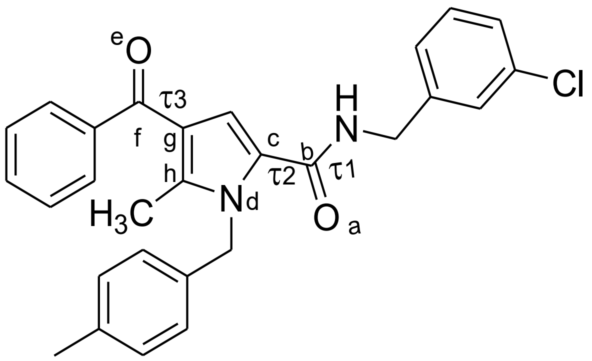

Family	ΔE_{GM}[a] (kcal/mol)	Torsional Angles (°)			Occurrence Rate (%)
		τ1[b]	τ2[c]	τ3[d]	
TTC	0.00-4.95	~180	~180	~0.00	24
TTT	0.03-4.91	~180	~180	~180	25
TCT	1.58-4.95	~180	~0.00	~180	25
TCC	2.39-4.94	~180	~0.00	~0.00	26

[a]The values reported refer to the lowest and the highest energy conformers of the family. [b] τ1 torsional angle is calculated considering the amide bond atoms. [c] τ2: a, b, c, and d atoms. [d] τ3: e, f, g, and h atoms.

Table S2. ΔE_{GM} values (kcal/mol), torsional angle values (degrees) and occurrence rates (%) of conformational families of **10a** considering MM conformers within 5 kcal/mol from the global minimum.

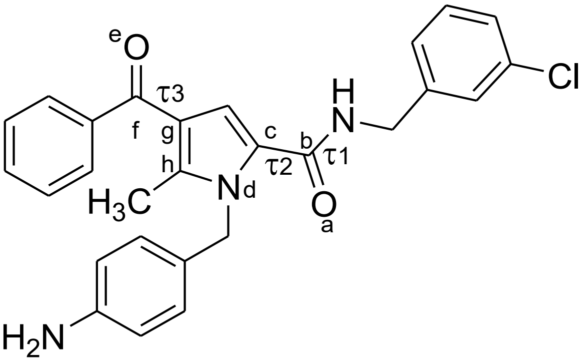

Family	ΔE_{GM}[a] (kcal/mol)	Torsional Angles (°) τ1[b]	τ2[c]	τ3[d]	Occurrence Rate (%)
TTC	0.00-4.97	~180	~180	~0.00	26
TTT	0.26-4.97	~180	~180	~180	23
TCT	1.90-4.87	~180	~0.00	~180	28
TCC	2.44-4.96	~180	~0.00	~0.00	23

[a]The values reported refer to the lowest and the highest energy conformers of the family. [b] τ1 torsional angle is calculated considering the amide bond atoms. [c] τ2: a, b, c, and d atoms. [d] τ3: e, f, g, and h atoms.

Table S3. ΔE_{GM} values (kcal/mol), torsional angle values (degrees) and occurrence rates (%) of conformational families of **9a** considering MM conformers within 5 kcal/mol from the global minimum.

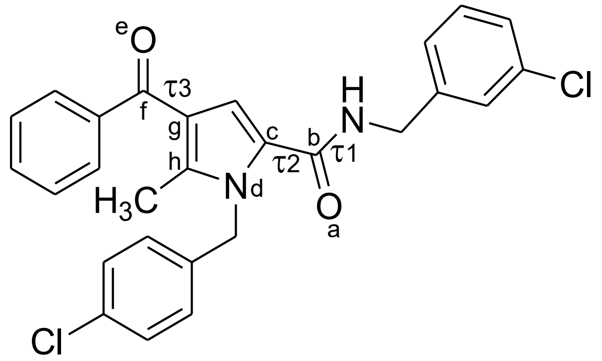

Family	ΔE_{GM}[a] (kcal/mol)	Torsional Angles (°) τ1[b]	τ2[c]	τ3[d]	Occurrence Rate (%)
TTC	0.00-4.88	~180	~180	~0.00	23
TTT	0.34-4.96	~180	~180	~180	25
TCT	1.96-4.89	~180	~0.00	~180	29
TCC	2.37-4.92	~180	~0.00	~0.00	23

[a]The values reported refer to the lowest and the highest energy conformers of the family. [b] τ1 torsional angle is calculated considering the amide bond atoms. [c] τ2: a, b, c, and d atoms. [d] τ3: e, f, g, and h atoms.

Table S4. ΔE_{GM} values (kcal/mol), torsional angle values (degrees) and occurrence rates (%) of conformational families of **9b** considering MM conformers within 5 kcal/mol from the global minimum.

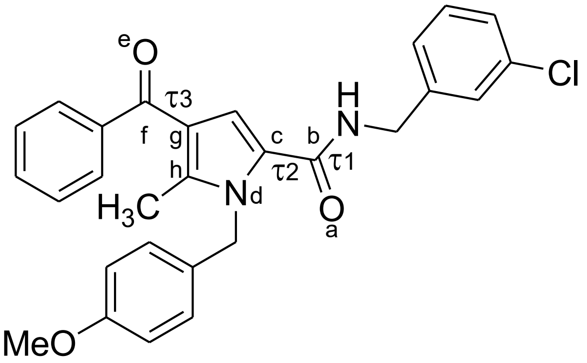

Family	ΔE_{GM}[a] (kcal/mol)	Torsional Angles (°)			Occurrence Rate (%)
		τ1[b]	τ2[c]	τ3[d]	
TTC	0.00-4.98	~180	~180	~0.00	23
TTT	0.45-4.98	~180	~180	~180	18
TCC	1.96-4.97	~180	~0.00	~0.00	34
TCT	2.18-4.77	~180	~0.00	~180	25

[a]The values reported refer to the lowest and the highest energy conformers of the family. [b] τ1 torsional angle is calculated considering the amide bond atoms. [c] τ2: a, b, c, and d atoms. [d] τ3: e, f, g, and h atoms.

Table S5. ΔE_{GM} values (kcal/mol), torsional angle values (degrees) and occurrence rates (%) of conformational families of **9c** considering MM conformers within 5 kcal/mol from the global minimum.

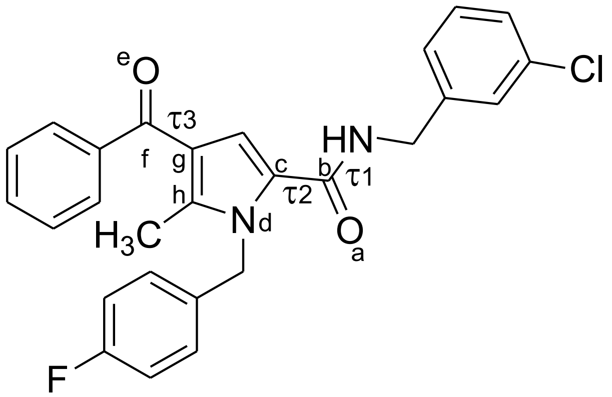

Family	ΔE_{GM}[a] (kcal/mol)	Torsional Angles (°) τ1[b]	τ2[c]	τ3[d]	Occurrence Rate (%)
TTC	0.00-4.99	~180	~180	~0.00	28
TTT	0.38-4.97	~180	~180	~180	21
TCT	1.84-4.20	~180	~0.00	~180	22
TCC	1.89-4.68	~180	~0.00	~0.00	29

[a]The values reported refer to the lowest and the highest energy conformers of the family. [b] τ1 torsional angle is calculated considering the amide bond atoms. [c] τ2: a, b, c, and d atoms. [d] τ3: e, f, g, and h atoms.

Table S6. ΔE_{GM} values (kcal/mol), torsional angle values (degrees) and occurrence rates (%) of conformational families of **9d** considering MM conformers within 5 kcal/mol from the global minimum.

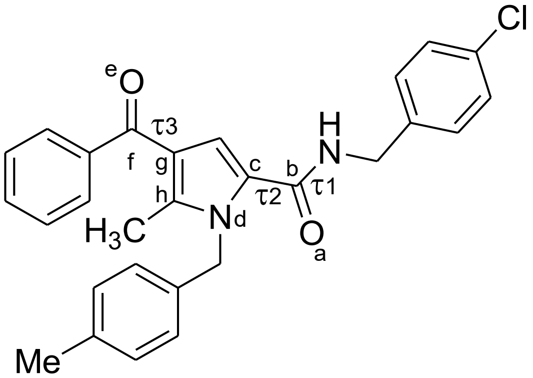

Family	ΔE_{GM}[a] (kcal/mol)	Torsional Angles (°)			Occurrence Rate (%)
		τ1[b]	τ2[c]	τ3[d]	
TTC	0.00-4.94	~180	~180	~0.00	26
TTT	0.05-4.72	~180	~180	~180	24
TCT	1.92-4.86	~180	~0.00	~180	22
TCC	2.02-4.58	~180	~0.00	~0.00	28

[a]The values reported refer to the lowest and the highest energy conformers of the family. [b] τ1 torsional angle is calculated considering the amide bond atoms. [c] τ2: a, b, c, and d atoms. [d] τ3: e, f, g, and h atoms.

Table S7. ΔE_{GM} values (kcal/mol), torsional angle values (degrees) and occurrence rates (%) of conformational families of **9e** considering MM conformers within 5 kcal/mol from the global minimum.

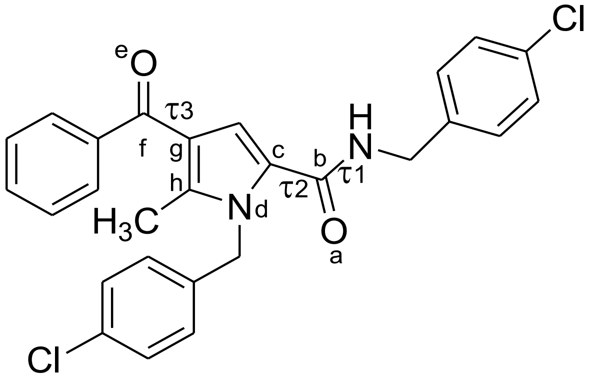

Family	ΔE_{GM}[a] (kcal/mol)	Torsional Angles (°) τ1[b]	τ2[c]	τ3[d]	Occurrence Rate (%)
TTT	0.00-4.82	~180	~180	~180	26
TTC	0.05-4.99	~180	~180	~0.00	22
TCC	1.64-4.58	~180	~0.00	~0.00	27
TCT	1.74-4.76	~180	~0.00	~180	25

[a]The values reported refer to the lowest and the highest energy conformers of the family. [b] τ1 torsional angle is calculated considering the amide bond atoms. [c] τ2: a, b, c, and d atoms. [d] τ3: e, f, g, and h atoms.

Table S8. ΔE_{GM} values (kcal/mol), torsional angle values (degrees) and occurrence rates (%) of conformational families of **9f** considering MM conformers within 5 kcal/mol from the global minimum.

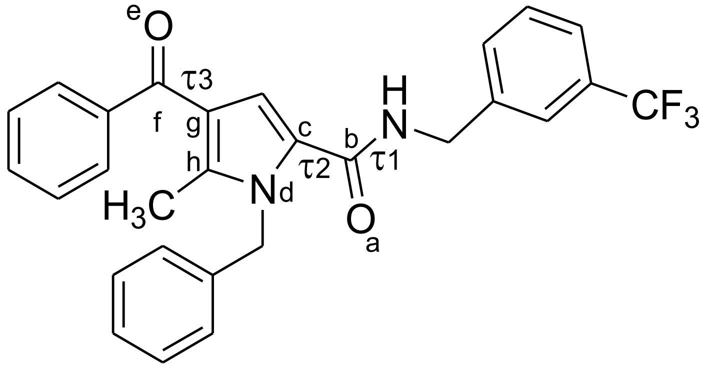

Family	ΔE_{GM}[a] (kcal/mol)	Torsional Angles (°)			Occurrence Rate (%)
		τ1[b]	τ2[c]	τ3[d]	
TTT	0.00-4.98	~180	~180	~180	22
TTC	0.33-4.99	~180	~180	~0.00	26
TCT	1.95-4.98	~180	~0.00	~180	24
TCC	2.37-4.90	~180	~0.00	~0.00	28

[a]The values reported refer to the lowest and the highest energy conformers of the family. [b] τ1 torsional angle is calculated considering the amide bond atoms. [c] τ2: a, b, c, and d atoms. [d] τ3: e, f, g, and h atoms.

Table S9. ΔE_{GM} values (kcal/mol), torsional angle values (degrees) and occurrence rates (%) of conformational families of **9g** considering MM conformers within 5 kcal/mol from the global minimum.

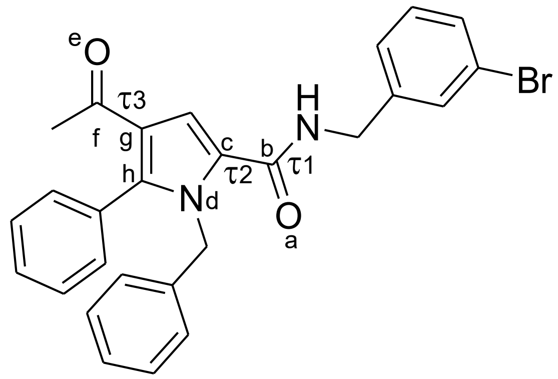

Family	ΔE_{GM}[a] (kcal/mol)	Torsional Angles (°) τ1[b]	τ2[c]	τ3[d]	Occurrence Rate (%)
TTC	0.00-4.98	~180	~180	~0.00	20
TTT	0.32-4.99	~180	~180	~180	24
TCC	2.57-4.99	~180	~0.00	~0.00	27
TCT	2.63-4.98	~180	~0.00	~180	29

[a]The values reported refer to the lowest and the highest energy conformers of the family. [b] τ1 torsional angle is calculated considering the amide bond atoms. [c] τ2: a, b, c, and d atoms.

Table S10. ΔE_{GM} values (kcal/mol), torsional angle values (degrees) and occurrence rates (%) of conformational families of **9h** considering MM conformers within 5 kcal/mol from the global minimum.

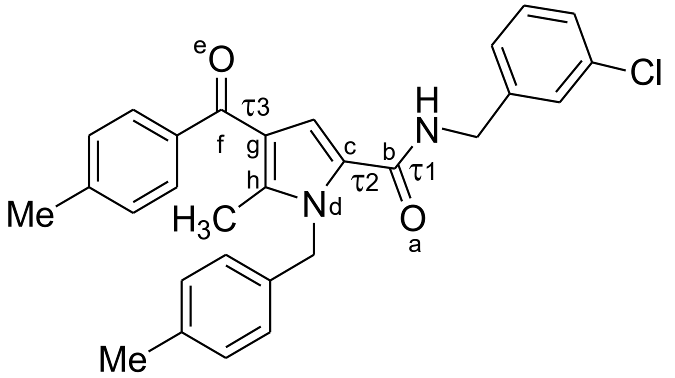

Family	ΔE_{GM}[a] (kcal/mol)	Torsional Angles (°)			Occurrence Rate (%)
		τ1[b]	τ2[c]	τ3[d]	
TTT	0.00-4.99	~180	~180	~180	25
TTC	0.05-4.94	~180	~180	~0.00	23
TCC	2.02-4.91	~180	~0.00	~0.00	29
TCT	2.19-4.92	~180	~0.00	~180	23

[a]The values reported refer to the lowest and the highest energy conformers of the family. [b] τ1 torsional angle is calculated considering the amide bond atoms. [c] τ2: a, b, c, and d atoms. [d] τ3: e, f, g, and h atoms.

Table S11. ΔE_{GM} values (kcal/mol), torsional angle values (degrees) and occurrence rates (%) of conformational families of **9i** considering MM conformers within 5 kcal/mol from the global minimum.

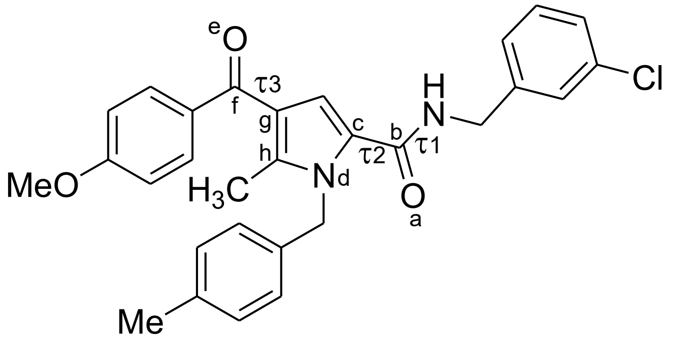

Family	ΔE_{GM}[a] (kcal/mol)	Torsional Angles (°)			Occurrence Rate (%)
		τ1[b]	τ2[c]	τ3[d]	
TTC	0.00-4.94	~180	~180	~0.00	25
TTT	0.15-4.98	~180	~180	~180	20
TCT	2.19-4.99	~180	~0.00	~180	26
TCC	2.38-4.87	~180	~0.00	~0.00	29

[a]The values reported refer to the lowest and the highest energy conformers of the family. [b] τ1 torsional angle is calculated considering the amide bond atoms. [c] τ2: a, b, c, and d atoms. [d] τ3: e, f, g, and h atoms.

Table S12. ΔE_{GM} values (kcal/mol), torsional angle values (degrees) and occurrence rates (%) of conformational families of **(R)-10b** considering MM conformers within 5 kcal/mol from the global minimum.

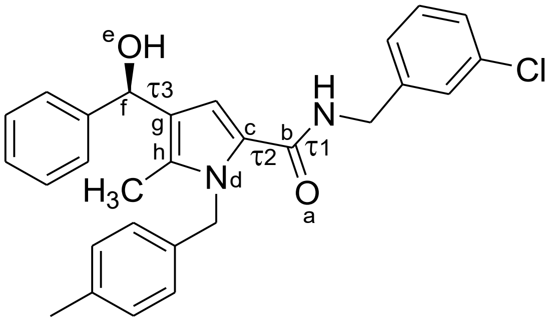

Family	ΔE_{GM}[a] (kcal/mol)	Torsional Angles (°)			Occurrence Rate (%)
		τ1[b]	τ2[c]	τ3[d]	
TTA$^-$	0.00-4.80	~180	~180	~-120	19
TTT	0.07-4.80	~180	~180	~180	21
TCT	1.76-4.96	~180	~0.00	~180	27.5
TTG$^+$	2.16-4.77	~180	~180	~60	12
TCA$^-$	2.75-4.37	~180	~0.00	~-120	19
TTG$^-$	3.36-3.66	~180	~180	~-60	1
TCG$^-$	4.88	~180	~0.00	~-60	0.5

[a]The values reported refer to the lowest and the highest energy conformers of the family. [b] τ1 torsional angle is calculated considering the amide bond atoms. [c] τ2: a, b, c, and d atoms. [d] τ2: e, f, g, and d htoms.

Table S13. Conformational energy, structural classification, and pharmacophore distances (Å) of calculated DFT conformers of **(R)-10b**.

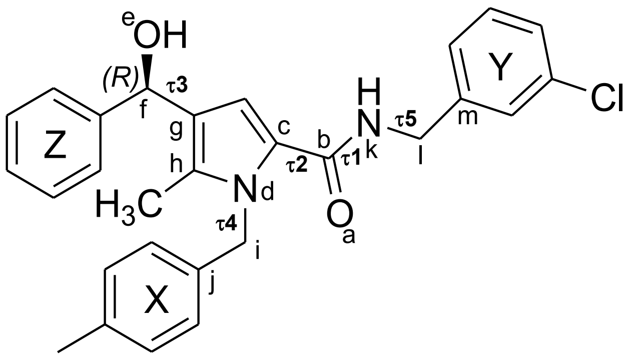

**Family**[a]	**Sub-family**	**ΔE$_{GM}$ (kcal/mol)**	**Distances (Å)**			**Torsional Angles (°)**[b-f]				
			d1 (X-Y)	d2 (X-Z)	d3 (Y-Z)	τ1	τ2	τ3	τ4	τ5
TCG^-	III	0.00	9.6	8.3	7.9	-173	23	-85	95	121
TCA^-	I	0.60	7.0	7.1	9.5	-172	23	-145	95	-103
TCG^+	III	0.89	7.3	7.0	10.3	174	-22	64	-94	112

[a] The data reported are referred to the enantiomer R, the enantiomer S presents the same conformational energy, the same pharmacophore distances, and the same absolute torsion angle values but with opposite sign. [b]τ1 torsional angle is calculated considering the amide bond atoms. [c]τ2: a, b, c, and d atoms. [d]τ3: e, f, g, and h atoms. [e]τ4: c, d, i, and j atoms. [f]τ5: b, k, l, and m atoms.

Table S14. Conformational energy, structural classification, and pharmacophore distances (Å) of calculated DFT conformers of **9g**.

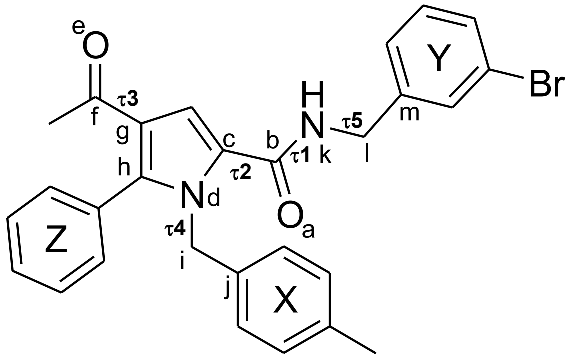

**Family**[a]	**Sub-family**	**ΔE$_{GM}$ (kcal/mol)**	**Distances (Å)**			**Torsional Angles (°)**[b-f]				
			d1 (X-Y)	d2 (X-Z)	d3 (Y-Z)	τ1	τ2	τ3	τ4	τ5
TCC	I	0.00	6.1	5.7	10.4	-173	27	-2	71	-107
TTC	IV	1.91	4.5	5.4	9.2	165	154	3	90	-176

[a] The data reported are referred to the enantiomer E1, the other enantiomer presents the same conformational energy, the same pharmacophore distances, and the same absolute torsion angle values but with opposite sign. [b]τ1 torsional angle is calculated considering the amide bond atoms. [c]τ2: a, b, c, and d atoms. [d]τ3: e, f, g, and h atoms. [e]τ4: c, d, i, and j atoms. [f]τ5: b, k, l, and m atoms.

Table S15. Conformational energy, structural classification, and intramolecular distances (Å) of calculated DFT conformers of **1**.

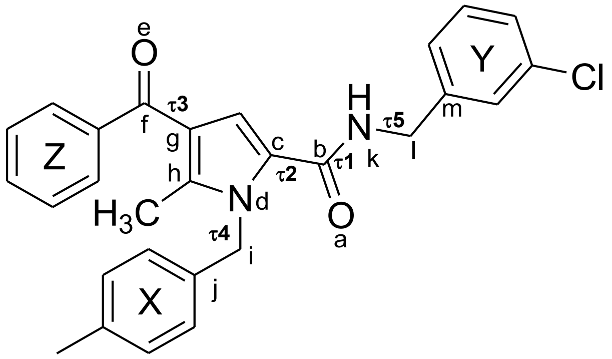

**Family**[a]	**Sub-family**	**ΔE$_{GM}$ (kcal/mol)**	**Distances (Å)**			**Torsional Angles (°)**[b-f]				
			d1 (X-Y)	d2 (X-Z)	d3 (Y-Z)	τ1	τ2	τ3	τ4	τ5
TCC	I	0.00	7.1	8.7	9.2	-173	25	-21	94	-111
TCC	II	0.57	6.1	9.3	10.1	-174	26	22	84	-84
TCT	I	1.66	7.5	7.2	11.0	-174	26	-148	101	-113
TCT	I	1.66	9.4	7.3	10.6	172	-24	-149	111	-124

[a] The data reported are referred to the enantiomer E1, the other enantiomer presents the same conformational energy, the same pharmacophore distances, and the same absolute torsion angle values but with opposite sign. [b]τ1 torsional angle is calculated considering the amide bond atoms. [c]τ2: a, b, c, and d atoms. [d]τ3: e, f, g, and h atoms. [e]τ4: c, d, i, and j atoms. [f]τ5: b, k, l, and m atoms.

Table S16. Conformational energy, structural classification, and intramolecular distances (Å) of calculated DFT conformers of **9a**.

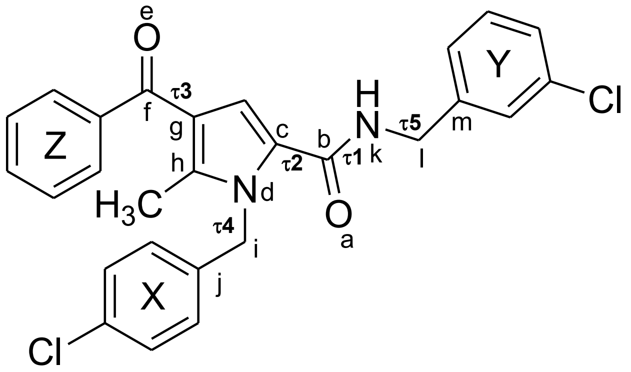

Family[a]	Sub-family	ΔE_{GM} (kcal/mol)	Distances (Å)			Torsional Angles (°)[b-f]				
			d1 (X-Y)	d2 (X-Z)	d3 (Y-Z)	τ1	τ2	τ3	τ4	τ5
TCC	I	0.00	7.0	8.7	9.3	-173	25	-21	91	-110
TCC	II	0.43	6.7	9.2	10.1	-174	25	22	98	-82
TCT	I	1.59	7.1	7.3	10.9	-172	26	-147	99	-105
TCT	III	1.67	9.6	7.3	10.8	-173	25	-147	98	120

[a] The data reported are referred to the enantiomer E1, the other enantiomer presents the same conformational energy, the same pharmacophore distances, and the same absolute torsion angle values but with opposite sign. [b]τ1 torsional angle is calculated considering the amide bond atoms. [c]τ2: a, b, c, and d atoms. [d]τ3: e, f, g, and h atoms. [e]τ4: c, d, i, and j atoms. [f]τ5: b, k, l, and m atoms.

Table S17. Conformational energy, structural classification, and intramolecular distances (Å) of calculated DFT conformers of **9b**.

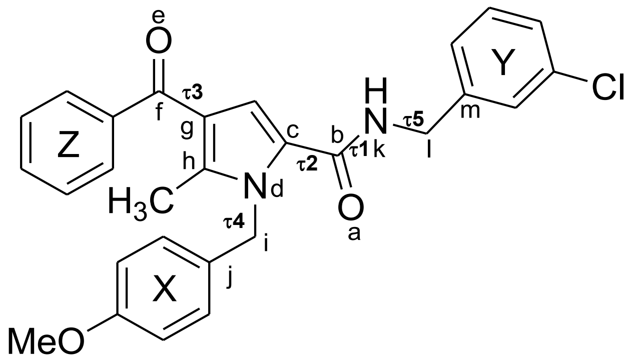

Family[a]	Sub-family	ΔEGM (kcal/mol)	Distances (Å)			Torsional Angles (°)[b-f]				
			d1 (X-Y)	d2 (X-Z)	d3 (Y-Z)	τ1	τ2	τ3	τ4	τ5
TCC	I	0.00	6.4	9.3	9.7	-173	28	22	78	-112
TCC	II	0.27	5.7	9.3	10.1	-174	26	22	76	-83
TCT	I	1.31	7.5	7.2	11.0	-174	26	-148	102	-112
TCT	III	1.38	9.7	7.2	10.9	-173	26	-148	102	120

[a] The data reported are referred to the enantiomer E1, the other enantiomer presents the same conformational energy, the same pharmacophore distances, and the same absolute torsion angle values but with opposite sign. [b]τ1 torsional angle is calculated considering the amide bond atoms. [c]τ2: a, b, c, and d atoms. [d]τ3: e, f, g, and h atoms. [e]τ4: c, d, i, and j atoms. [f]τ5: b, k, l, and m atoms.

Table S18. Conformational energy, structural classification, and intramolecular distances (Å) of calculated DFT conformers of **9c**.

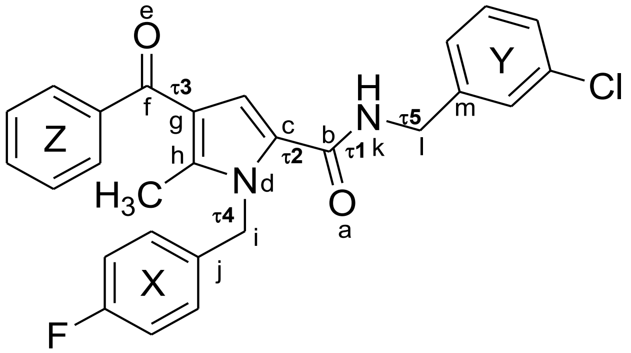

**Family**[a]	**Sub-family**	**ΔE$_{GM}$ (kcal/mol)**	**Distances (Å)**			**Torsional Angles (°)**[b-f]				
			d1 (X-Y)	d2 (X-Z)	d3 (Y-Z)	τ1	τ2	τ3	τ4	τ5
TCC	I	0.00	6.5	9.3	9.6	-174	26	22	79	-115
TCC	II	0.24	5.9	9.3	10.1	-174	26	22	78	-85
TCT	I	1.33	7.5	7.2	10.9	-175	25	-147	101	-116
TCT	III	1.42	9.7	7.3	10.8	-173	26	-147	101	124

[a] The data reported are referred to the enantiomer E1, the other enantiomer presents the same conformational energy, the same pharmacophore distances, and the same absolute torsion angle values but with opposite sign. [b]τ1 torsional angle is calculated considering the amide bond atoms. [c]τ2: a, b, c, and d atoms. [d]τ3: e, f, g, and h atoms. [e]τ4: c, d, i, and j atoms. [f]τ5: b, k, l, and m atoms.

Table S19. Conformational energy, structural classification, and intramolecular distances (Å) of calculated DFT conformers of **9d**.

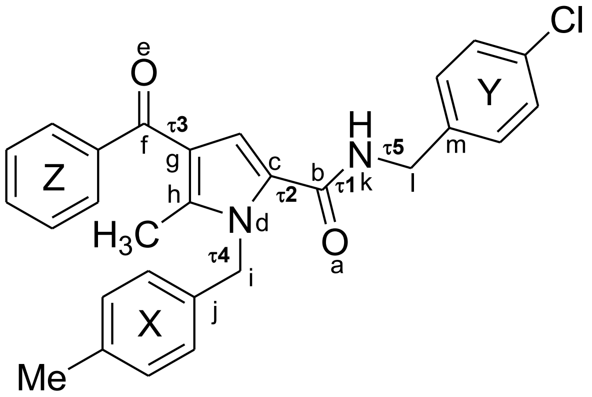

Family[a]	Sub-family	ΔE_{GM} (kcal/mol)	Distances (Å)			Torsional Angles (°)[b-f]				
			d1 (X-Y)	d2 (X-Z)	d3 (Y-Z)	τ1	τ2	τ3	τ4	τ5
TCC	I	0.00	7.2	9.2	9.7	-173	27	22	99	-111
TCC	II	0.29	6.7	9.2	10.1	-174	26	22	99	-82
TCT	I	1.46	7.4	7.3	10.9	-174	26	-147	100	-113
TCT	III	1.50	9.7	7.3	10.7	-173	26	-147	100	120

[a] The data reported are referred to the enantiomer E1, the other enantiomer presents the same conformational energy, the same pharmacophore distances, and the same absolute torsion angle values but with opposite sign. [b]τ1 torsional angle is calculated considering the amide bond atoms. [c]τ2: a, b, c, and d atoms. [d]τ3: e, f, g, and h atoms. [e]τ4: c, d, i, and j atoms. [f]τ5: b, k, l, and m atoms.

Table S20. Conformational energy, structural classification, and intramolecular distances (Å) of calculated DFT conformers of **9e**.

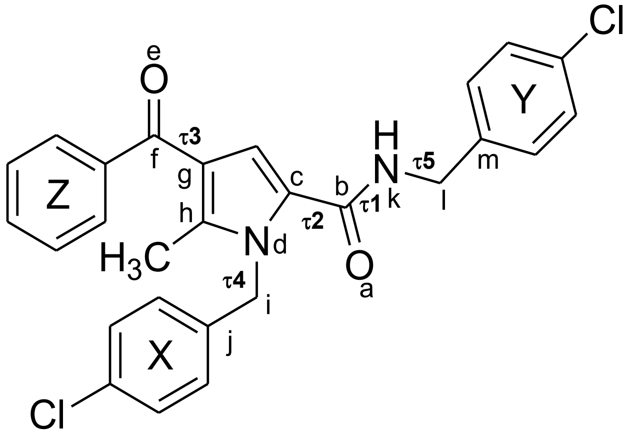

Family[a]	Sub-family	ΔE$_{GM}$ (kcal/mol)	Distances (Å)			Torsional Angles (°)[b-f]				
			d1 (X-Y)	d2 (X-Z)	d3 (Y-Z)	τ1	τ2	τ3	τ4	τ5
TCC	I	0.00	6.8	9.2	9.7	-173	26	22	87	-112
TCC	II	0.18	6.7	9.2	10.1	-174	26	22	98	-85
TCT	I	1.38	7.3	7.3	10.8	-173	26	-147	100	-112
TCT	III	1.40	9.5	7.3	10.7	-172	23	-147	99	131

[a] The data reported are referred to the enantiomer E1, the other enantiomer presents the same conformational energy, the same pharmacophore distances, and the same absolute torsion angle values but with opposite sign. [b]τ1 torsional angle is calculated considering the amide bond atoms. [c]τ2: a, b, c, and d atoms. [d]τ3: e, f, g, and h atoms. [e]τ4: c, d, i, and j atoms. [f]τ5: b, k, l, and m atoms.

Table S21. Conformational energy, structural classification, and intramolecular distances (Å) of calculated DFT conformers of **9f**.

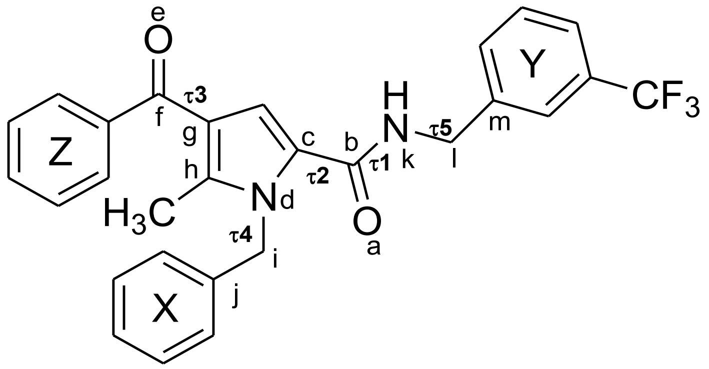

Family[a]	Sub-family	ΔEGM (kcal/mol)	Distances (Å)			Torsional Angles (°)[b-f]				
			d1 (X-Y)	d2 (X-Z)	d3 (Y-Z)	τ1	τ2	τ3	τ4	τ5
TCC	I	0.00	7.3	9.2	9.8	-174	26	22	99	-110
TCC	II	0.31	6.3	9.2	10.2	-177	27	24	88	-81
TCT	I	1.44	7.1	7.3	10.9	-172	27	-147	100	-103
TCT	I	1.57	9.3	7.3	10.5	172	-24	-148	111	-121

[a] The data reported are referred to the enantiomer E1, the other enantiomer presents the same conformational energy, the same pharmacophore distances, and the same absolute torsion angle values but with opposite sign. [b]τ1 torsional angle is calculated considering the amide bond atoms. [c]τ2: a, b, c, and d atoms. [d]τ3: e, f, g, and h atoms. [e]τ4: c, d, i, and j atoms. [f]τ5: b, k, l, and m atoms.

Table S22. Conformational energy, structural classification, and intramolecular distances (Å) of calculated DFT conformers of **9h**.

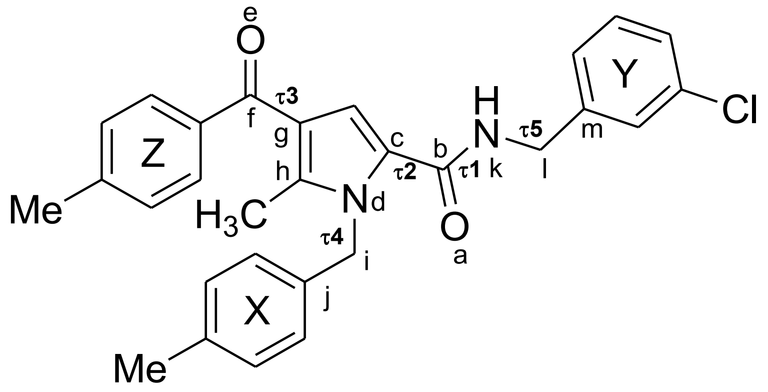

**Family**[a]	**Sub-family**	**ΔE$_{GM}$ (kcal/mol)**	**Distances (Å)**			**Torsional Angles (°)**[b-f]				
			d1 (X-Y)	d2 (X-Z)	d3 (Y-Z)	τ1	τ2	τ3	τ4	τ5
TCC	I	0.00	7.3	9.2	9.8	-173	27	24	99	-110
TCC	II	0.40	6.2	9.3	10.1	-174	26	23	84	-84
TCT	I	1.40	7.2	7.3	10.9	-172	27	-145	100	-105
TCT	I	1.44	9.3	7.3	10.6	173	-23	-147	110	-122

[a] The data reported are referred to the enantiomer E1, the other enantiomer presents the same conformational energy, the same pharmacophore distances, and the same absolute torsion angle values but with opposite sign. [b]τ1 torsional angle is calculated considering the amide bond atoms. [c]τ2: a, b, c, and d atoms. [d]τ3: e, f, g, and h atoms. [e]τ4: c, d, i, and j atoms. [f]τ5: b, k, l, and m atoms.

Table S23. Conformational energy, structural classification, and intramolecular distances (Å) of calculated DFT conformers of **9i**.

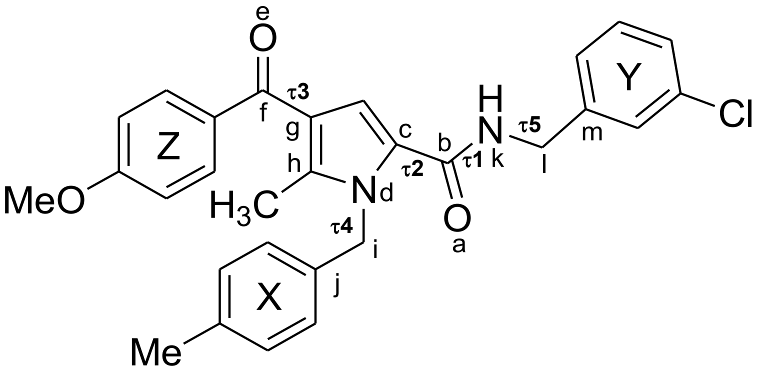

Family[a]	Sub-family	ΔE_{GM} (kcal/mol)	Distances (Å)			Torsional Angles (°)[b-f]				
			d1 (X-Y)	d2 (X-Z)	d3 (Y-Z)	τ1	τ2	τ3	τ4	τ5
TCC	I	0.00	7.4	9.2	9.8	-174	26	26	98	-114
TCC	II	0.23	6.1	9.3	10.2	-174	26	26	84	-84
TCT	I	1.21	7.4	7.2	10.9	-174	26	-143	100	-112
TCT	I	1.30	9.4	7.3	10.5	172	-24	-145	110	-125

[a] The data reported are referred to the enantiomer E1, the other enantiomer presents the same conformational energy, the same pharmacophore distances, and the same absolute torsion angle values but with opposite sign. [b]τ1 torsional angle is calculated considering the amide bond atoms. [c]τ2: a, b, c, and d atoms. [d]τ3: e, f, g, and h atoms. [e]τ4: c, d, i, and j atoms. [f]τ5: b, k, l, and m atoms.

Table S24. TSP conformers grouped according to the intramolecular distances between the X, Y, and Z rings.

.

Group	Conformer	Compounds	Distances (Å)[a]			Pharmacophore[b]
			d1 (X-Y)	d2 (X-Z)	d3 (Y-Z)	
A	TCC I	**1, 9a-f, 9h-i** and **10a**	7.0	9.1	9.6	1
B	TCC II	**1, 9a-f, 9h-i** and **10a**	6.2	9.3	10.1	-
C	TCT I	**1, 9a-f, 9h-i** and **10a**	7.3	7.2	10.9	2
C	TCA^-	**(±)-10b**	7.0	7.1	9.5	2
C	TCG^+	**(±)-10b**	7.3	7.0	10.3	2
D	TCT I	**1, 9d, 9h, 9i**	9.3	7.3	10.5	-
E	TCT III	**9a-c, 9e-f** and **10a**	9.7	7.3	10.8	-
F	TCC I	**9g**	6.1	5.6	10.4	3
G	TTC IV	**9g**	4.5	5.4	9.2	-
H	TCG^-	**(±)-10b**	9.6	8.3	7.9	4

[a]Average values. [b]Conformers matching similarity criteria with the intra-molecular distances of the hot spot residues of hydrophobic PPI motifs.

Table S25. Distances between the centroids of the side chains of the hot-spot residues of the considered PPI motifs.

Motifs	PDB ID	Distances (Å)					
		i,i+3	*i,i+4*	*i+3, i+4*	*i,i+7*	*i+4,i+7*	*i+3, i+7*
^{23}VxxLxxxV^{30}(Parallel)	2ZTA	6.6	-	-	10.1	-	7.7
^{14}LxxxLxxL^{21}(Antiparallel)	1GRJ	-	7.6	-	10.2	6.8	-
^{345}LxxLL^{349}	2J7X	6.9	5.2	7.2	-	-	-

Table S26. Calculated RMSD (Å) by fitting the centroids of the aromatic rings X, Y, and Z of the DFT conformers of TSPs on the centroids of the side chains of the considered PPI motif.

**Pharmacophore**	**Cmpd**	**Conformer**	**RMSD (Å)[a]**			
			i i+3 i+7		*i i+4 i+7*	
			**^{23}VxxLxxxV^{30} Parallel coiled coil PDB ID: 2ZTA**		**^{14}LxxxLxxL^{21} Antiparallel coiled coil PDB ID: 1GRJ**	
1	**10a**	TCC I	1.94	**2.25**	**2.18**	1.95
2	**10a**	TCT I	1.80	**2.08**	**1.65**	2.12
3	**9g**	TCC I	2.15	**2.42**	**2.35**	2.40
4	**10b**	TCG^{-}	1.99	**1.82**	**1.74**	2.25

[a]The RMSD values calculated for the conformational enantiomer E2 or for the configurational enantiomer S of **10b** are evidenced in bold

Table S27. Calculated RMSD (Å) by fitting the centroids of the aromatic rings X, Y, and Z of the selected DFT conformers on the centroids of the side chains of the considered PPI motif on human p14^{ARF}.

**Pharmacophore**	**Cmpd**	**Sub-fam**	**RMSD (Å)**			
			^{20}**V**RV**F**VVH**I**^{27}		^{20}**V**RVF**V**VH**I**^{27}	
			i, i+3, i+7		i, i+4, i+7	
1	**10a**	TCC I	2.27	**1.90**	**2.38**	2.55
2	**10a**	TCT I	1.62	**1.47**	**1.99**	2.34
3	**9g**	TCC I	2.51	**2.11**	**2.08**	2.03
4	**10b**	TCG^{-}	1.63	**1.93**	**2.45**	2.06

[a]The RMSD values calculated for the conformational enantiomer E2 or for the configurational enantiomer S of **10b** are evidenced in bold

Table S28. $cLogD_{7.4}$ of compounds **1**, **9a-i,** and **10a-b**.

Cpd	clog $D_{7.4}$
**1**	5.03
**9a**	5.63
**9b**	5.29
**9c**	4.65
**9d**	4.97
**9e**	5.29
**9f**	4.78
**9g**	5.29
**9h**	5.98
**9i**	4.77
**10a**	4.07
**(±) 10b**	5.31
**Nut-3a**	5.00

[a]ACD/Percepta 2017 software.

Synthesis

Scheme S1.

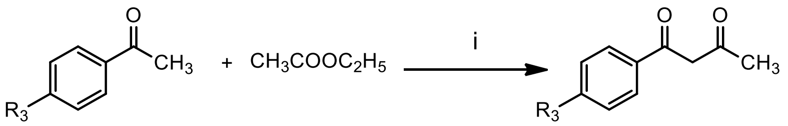

11a, R_3= CH_3
11b, R_3= OCH_3

5b, R_3= CH_3
5c, R_3= OCH_3

Reagents and conditions: *i) NaH (60 % dispersion in mineral oil), dry DMF, rt, 3h, then 60°C, 1h.*

1-(4-Methylphenyl)butane-1,3-dione (**5b)**

To a solution of **11a** (0.350 g, 2.61 mmol) in dry ethyl acetate (2 mL) was added a suspension of NaH (0.157 g, 3.92 mmol, 60% dispersion in mineral oil) in dry DMF (3 mL). The reaction mixture was stirred at room temperature for 3h and then heated at 60 °C until no starting material was detected by TLC (7:3 *n*-hexane/EtOAc, ~ 1h). Solvent was removed, and after cooling to 0 °C the resulting mixture was acidified with 3M HCl to give a solid product which was washed with H_2O and recrystallized (H_2O/EtOH) to furnish the title compound **5b** as a pale yellow solid. Yield: 73%. ^{1}H NMR (300 MHz, $CDCl_3$) (enol form) δ: 2.22 (s, 3H), 2.44 (s, 3H), 6.19 (s, 1H), 7.28 (d, 2H, *J*= 8.0 Hz), 7.81 (d, 2H, *J*= 8.0 Hz).

1-(4-Methoxyphenyl)butane-1,3-dione (**5c**)

Starting from **11b** (0.280 g, 1.86 mmol) and following the same procedure reported for **5b**, the tile compound was obtained as a white solid. Yield: 68%. ^{1}H NMR ($CDCl_3$) (enol form) δ: 2.19 (s, 3H), 3.88 (s, 3H), 6.13 (s,1H), 6.97 (d, 2H, *J*= 8.9 Hz), 7.89 (d, 2H, *J*= 8.8 Hz), 16.31 (s, 1H).

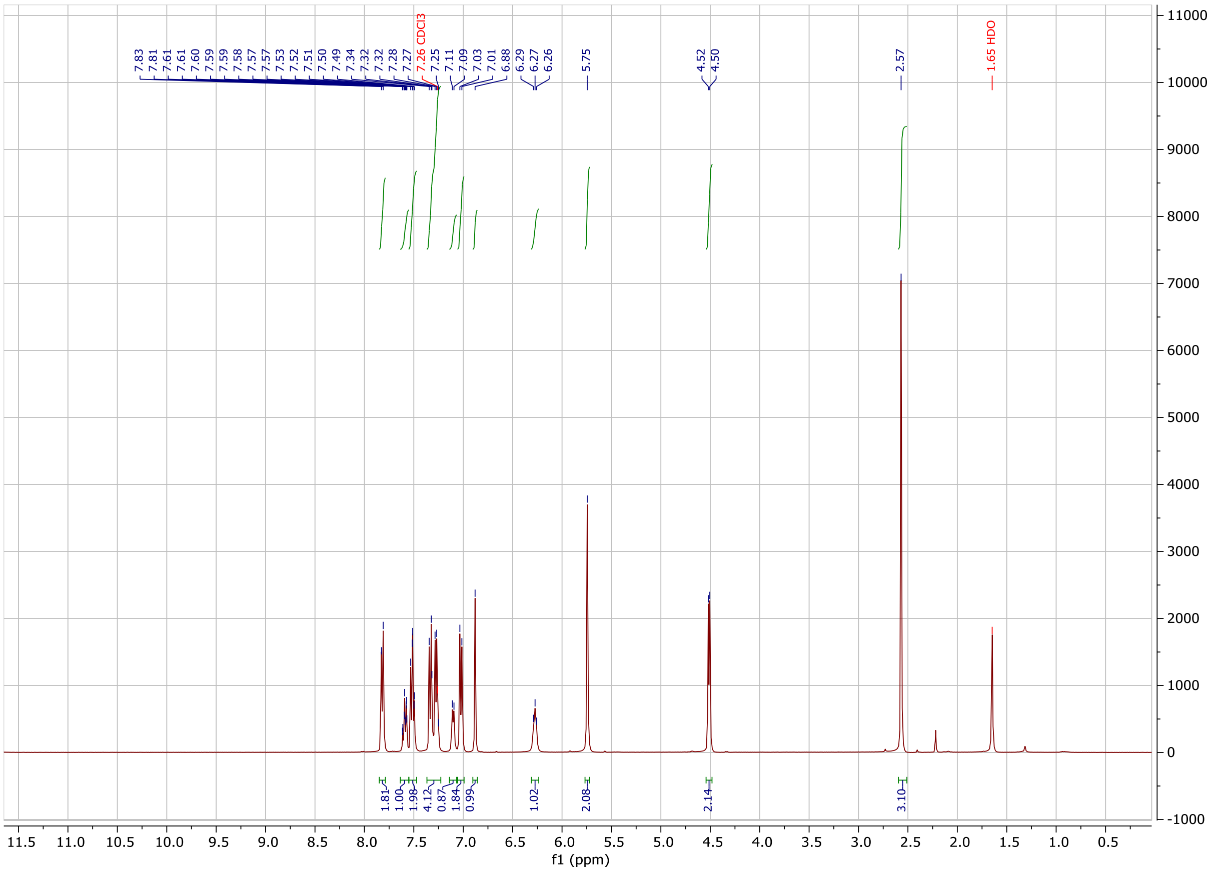

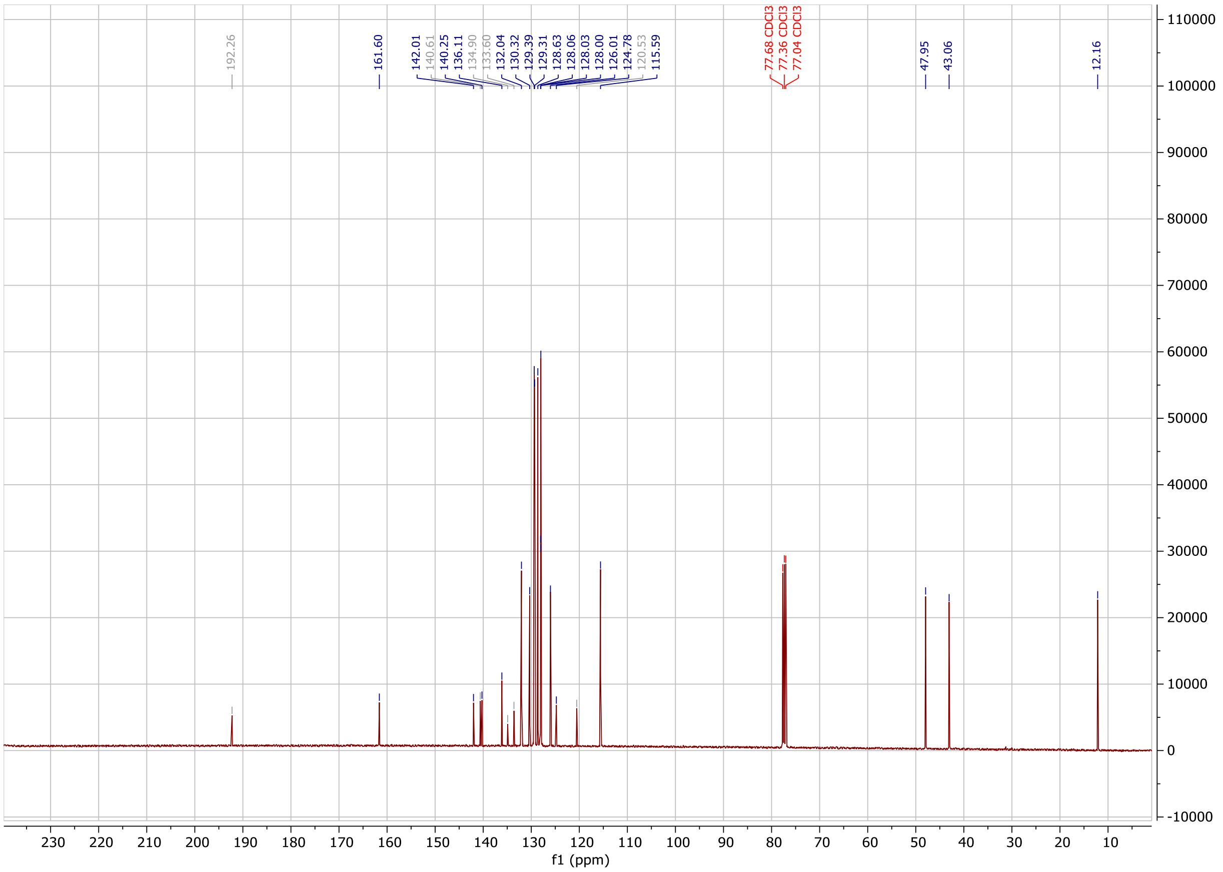

^{1}H NMR (400 MHz, $CDCl_3$) and ^{13}C NMR (101 MHz, $CDCl_3$) of compound **9a**

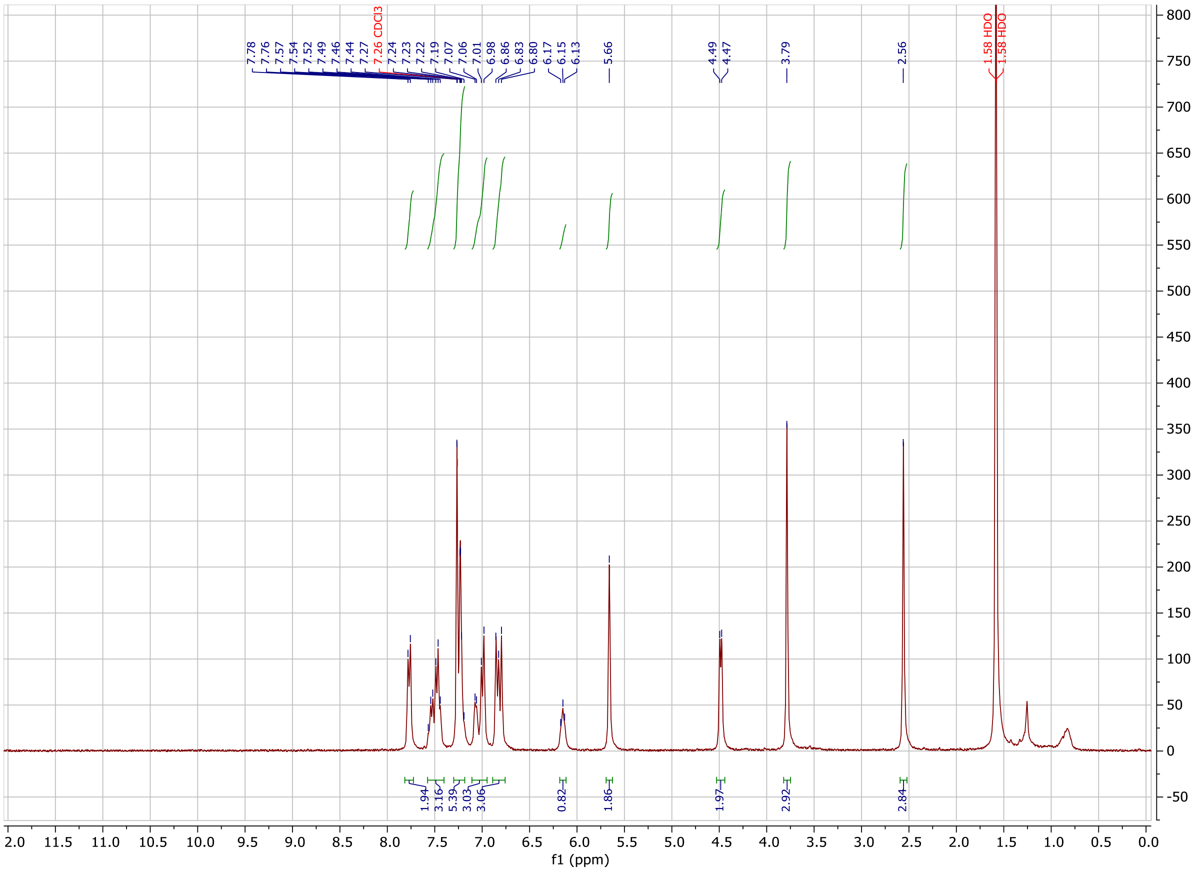

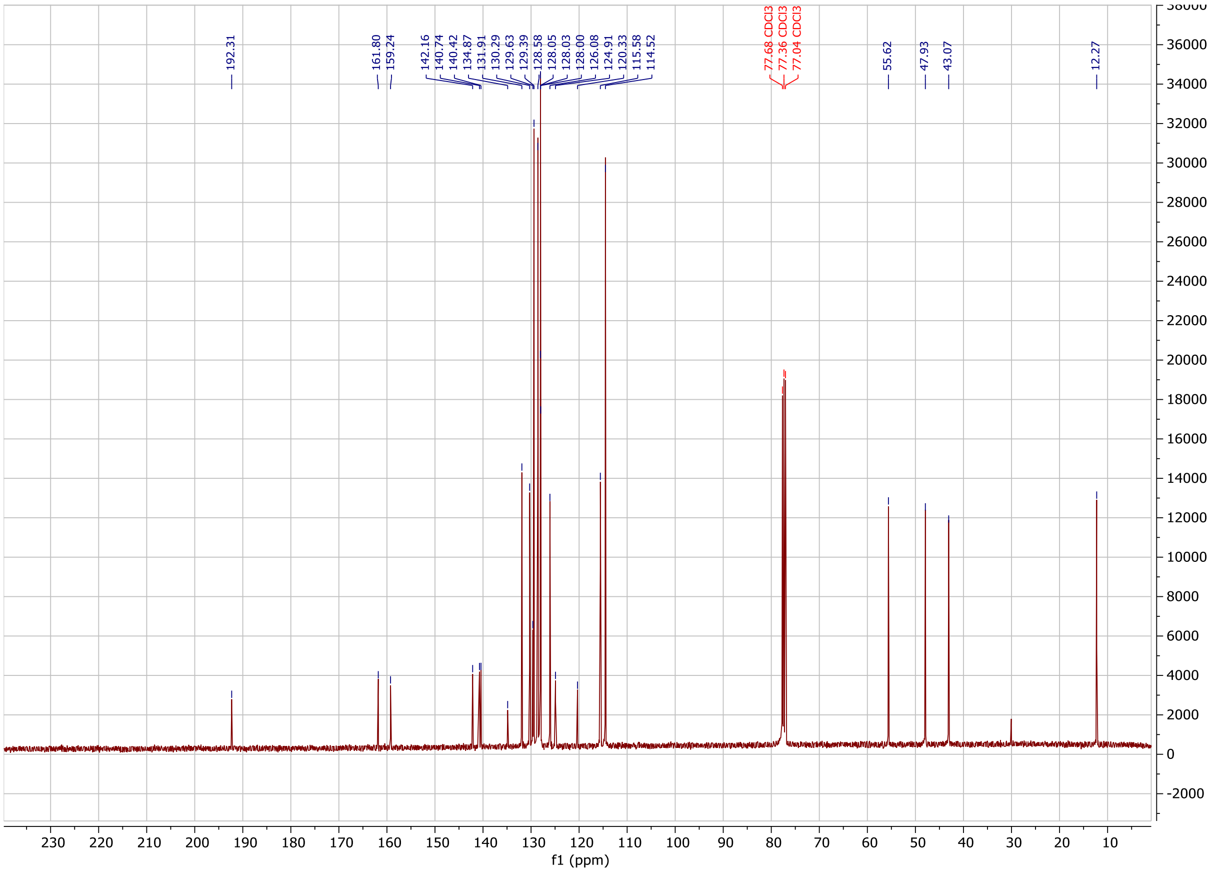

^{1}H NMR (300 MHz, $CDCl_3$) and ^{13}C NMR (75 MHz, $CDCl_3$) of compound **9b**

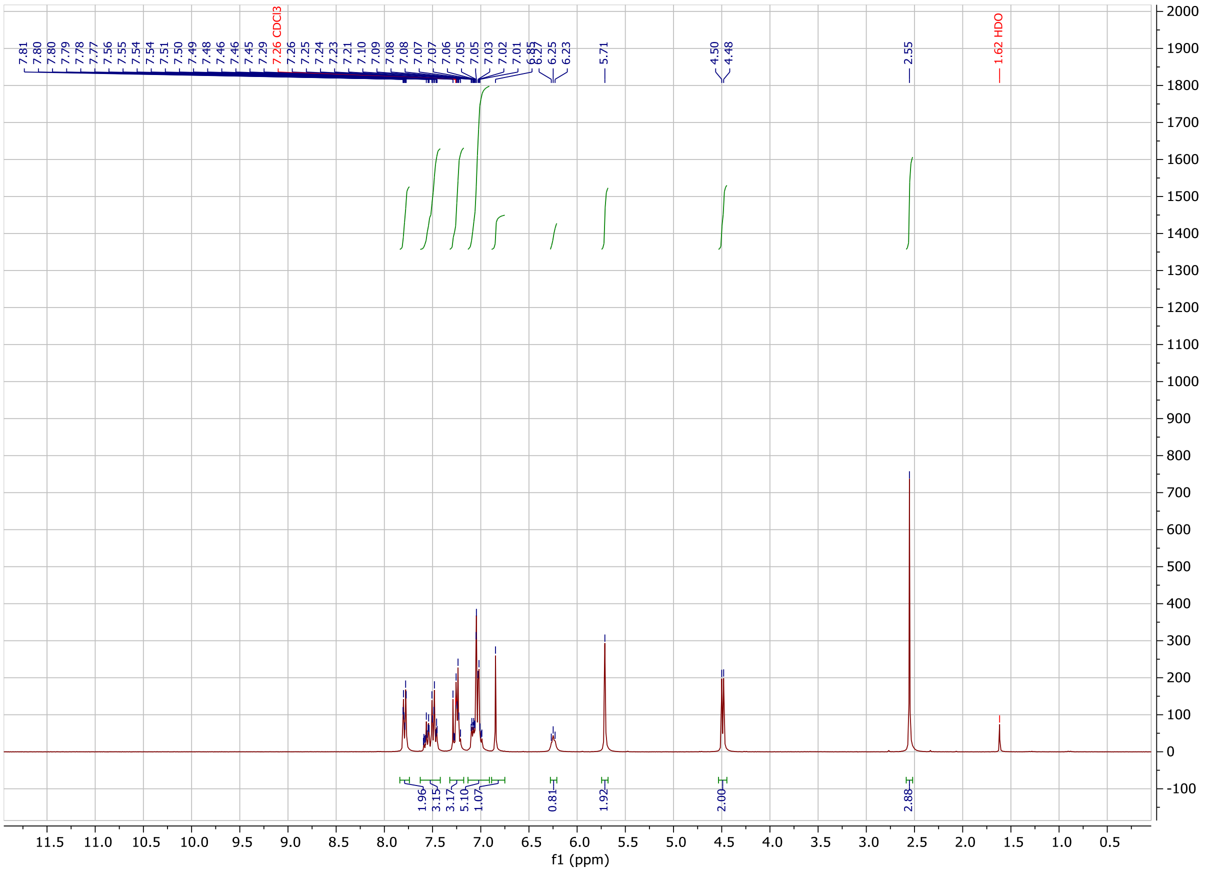

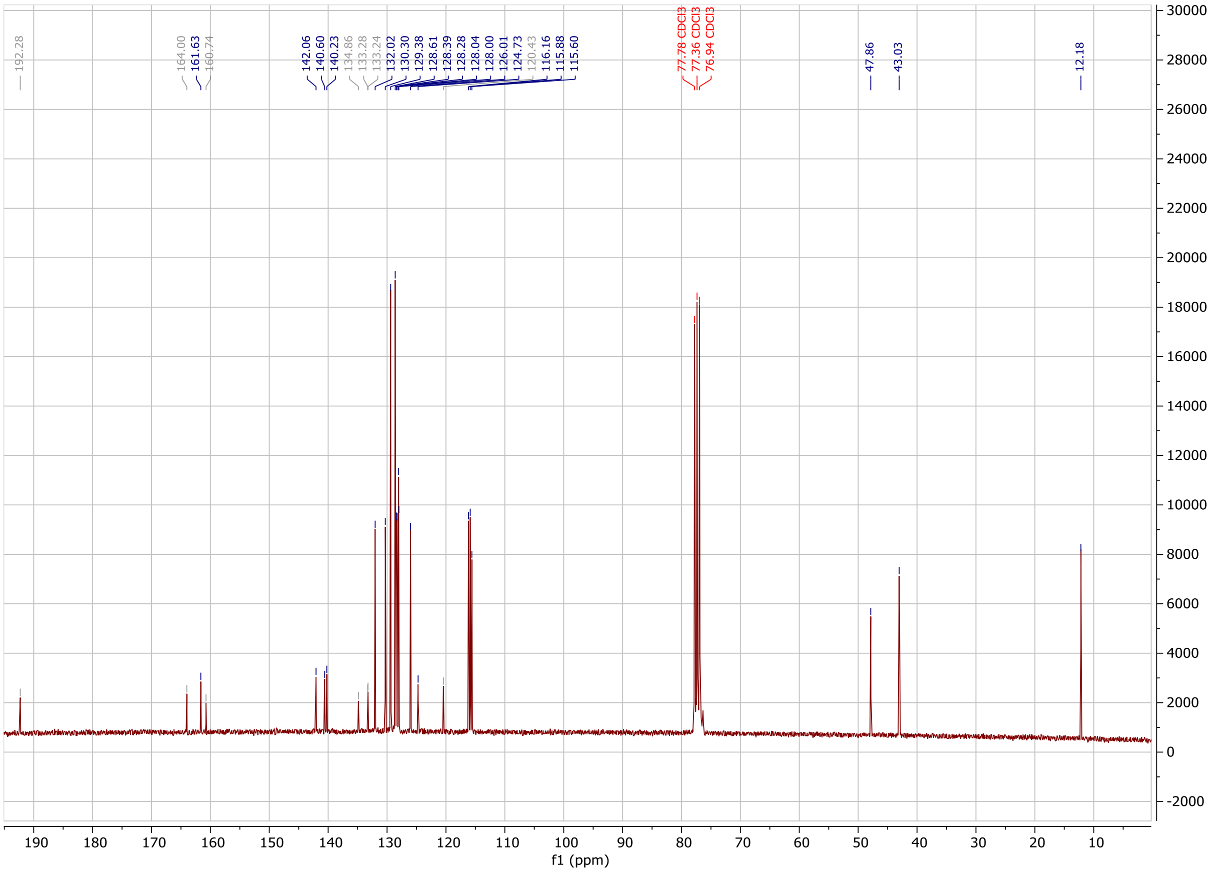

^{1}H NMR (300 MHz, $CDCl_3$) and ^{13}C NMR (75 MHz, $CDCl_3$) of compound **9c**

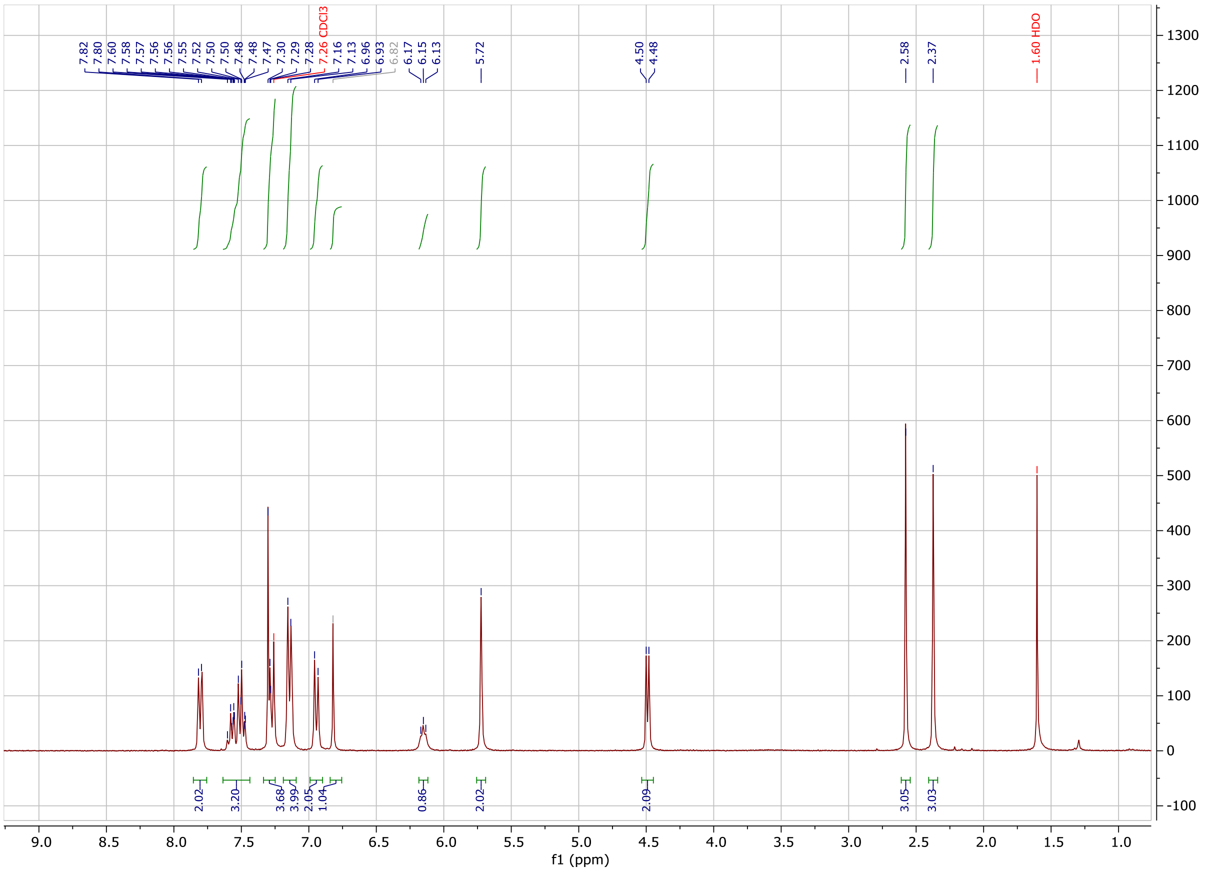

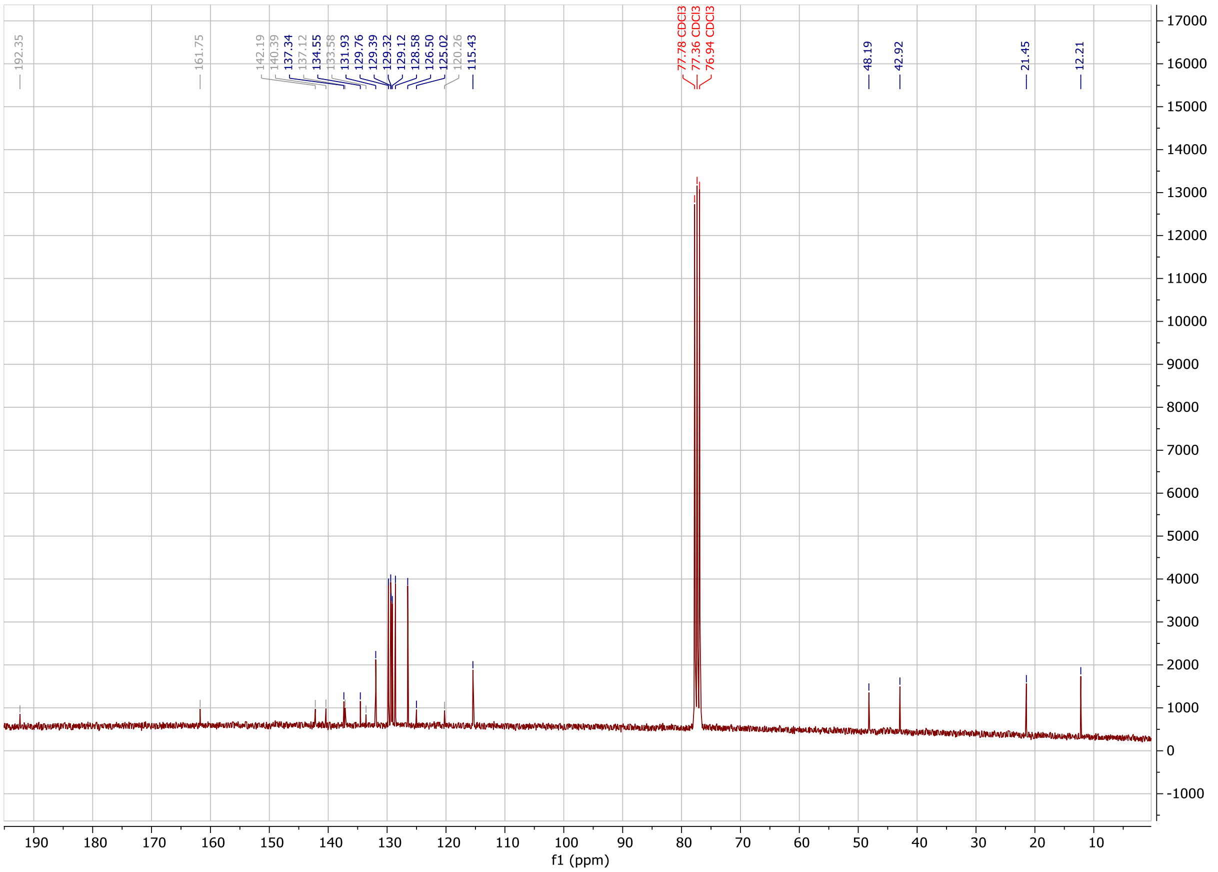

^{1}H NMR (300 MHz, $CDCl_3$) and ^{13}C NMR (75 MHz, $CDCl_3$) of compound **9d**

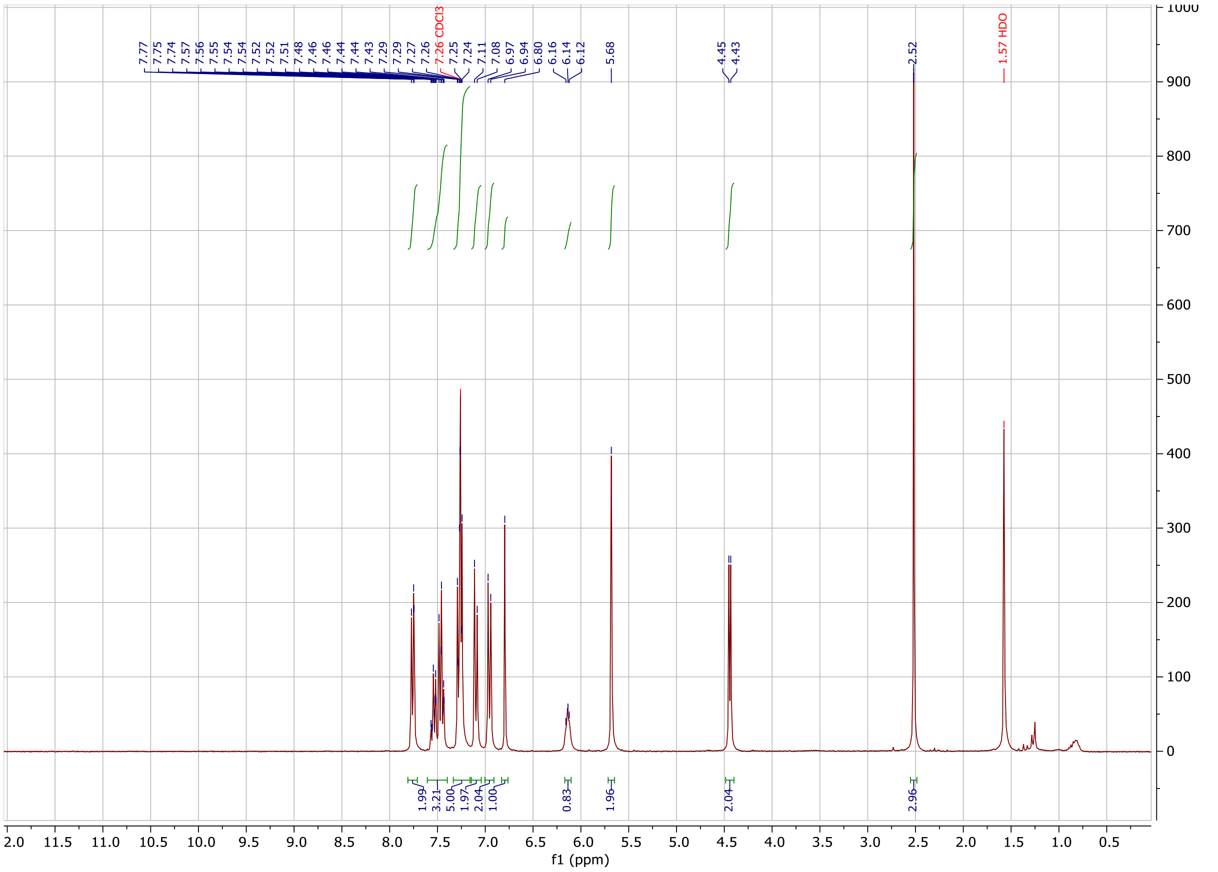

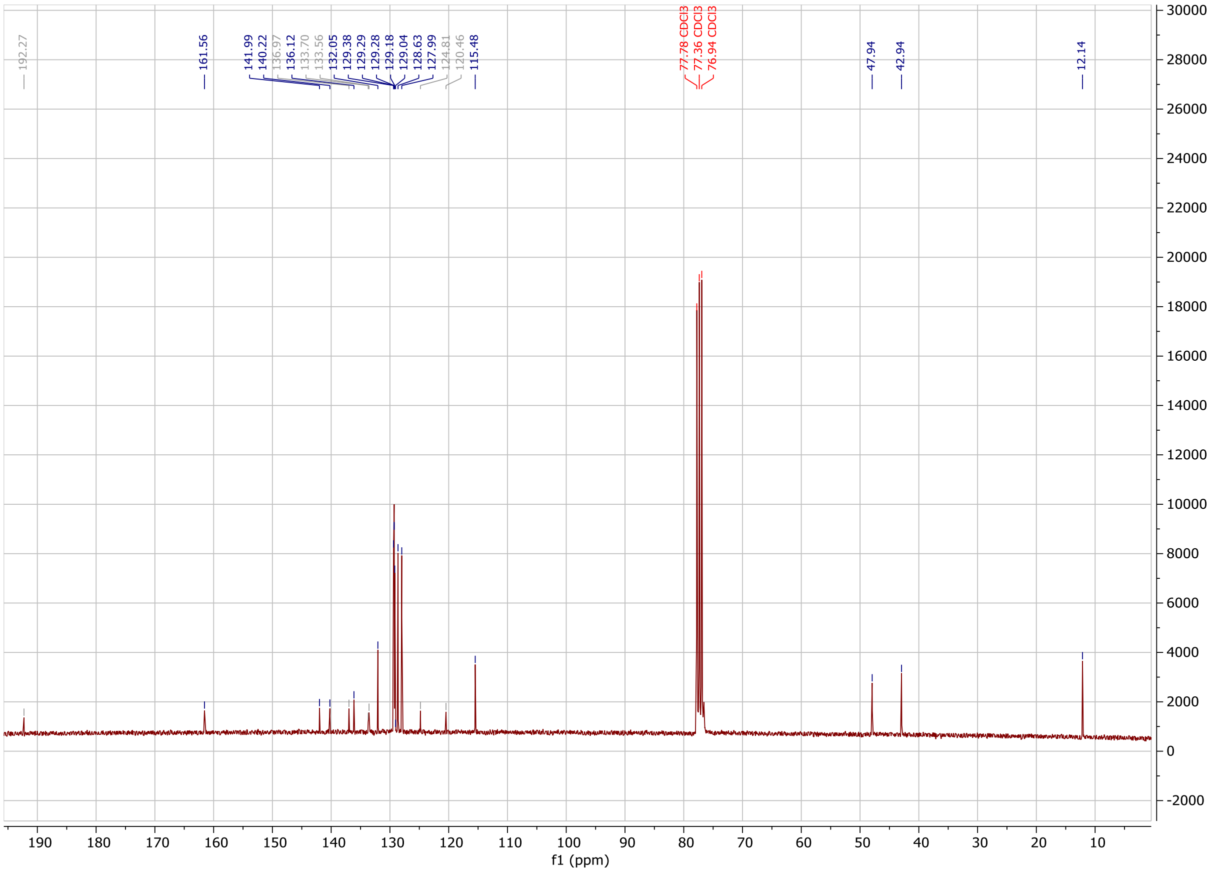

^{1}H NMR (300 MHz, $CDCl_3$) and ^{13}C NMR (75 MHz, $CDCl_3$) of compound **9e**

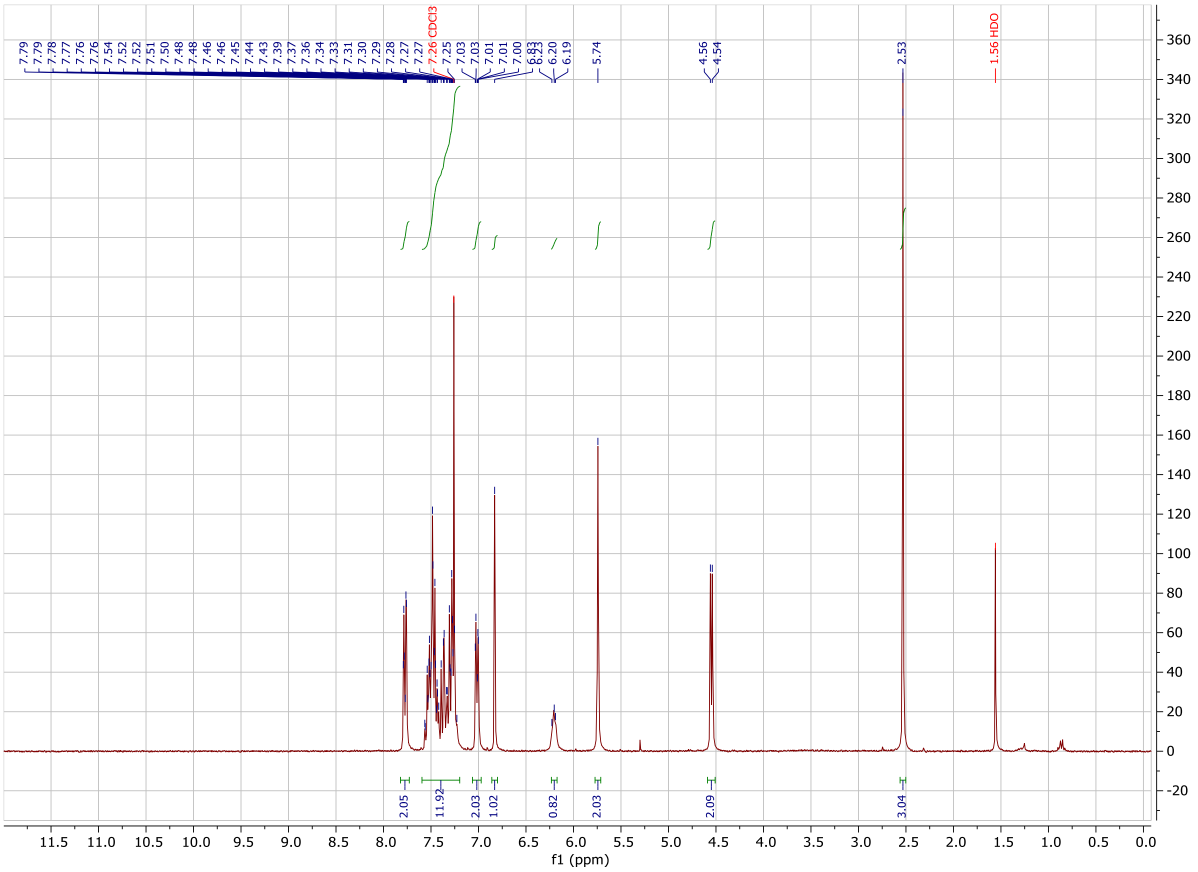

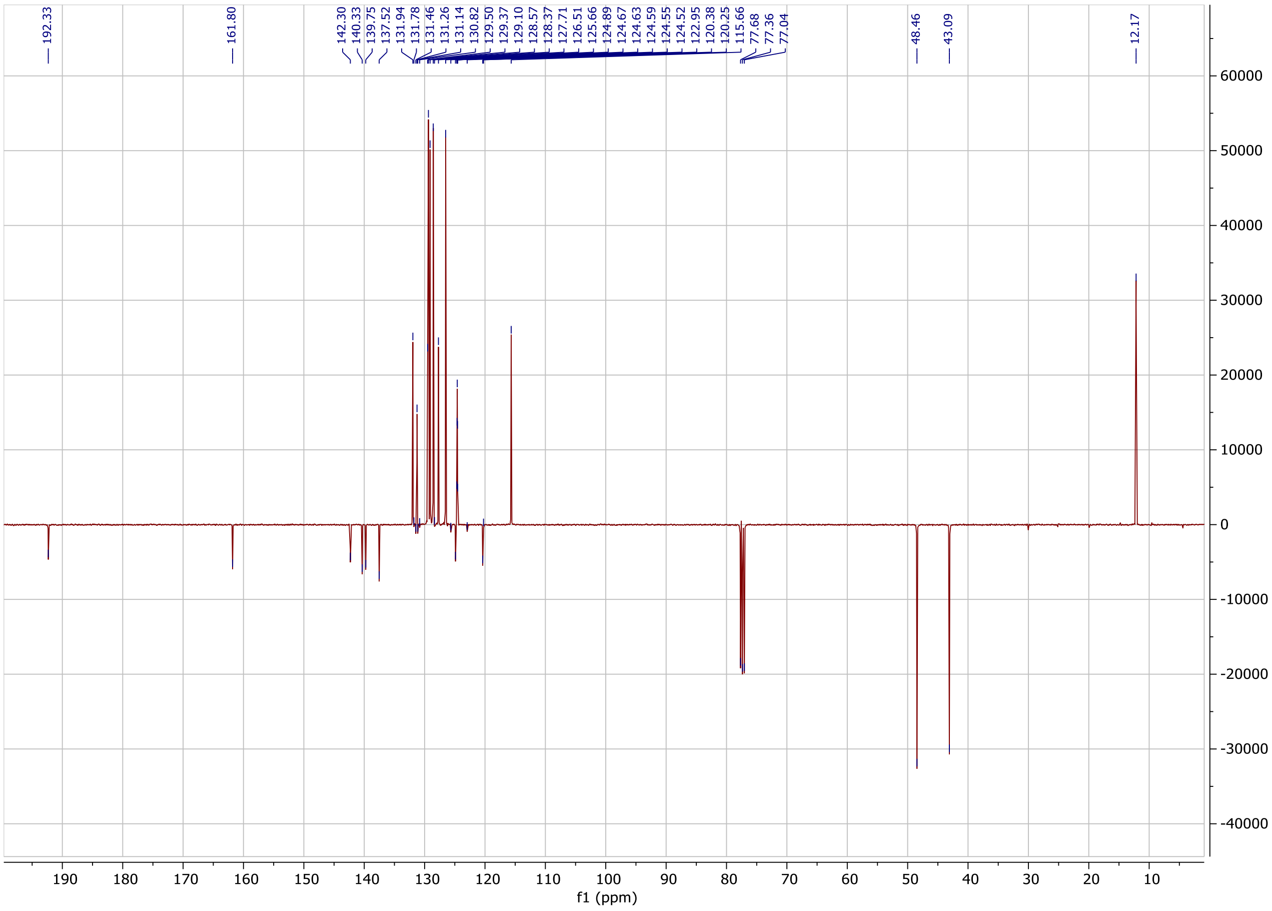

^{1}H NMR (400 MHz, $CDCl_3$) and ^{13}C NMR DEPT-q (101 MHz, $CDCl_3$) of compound **9f**

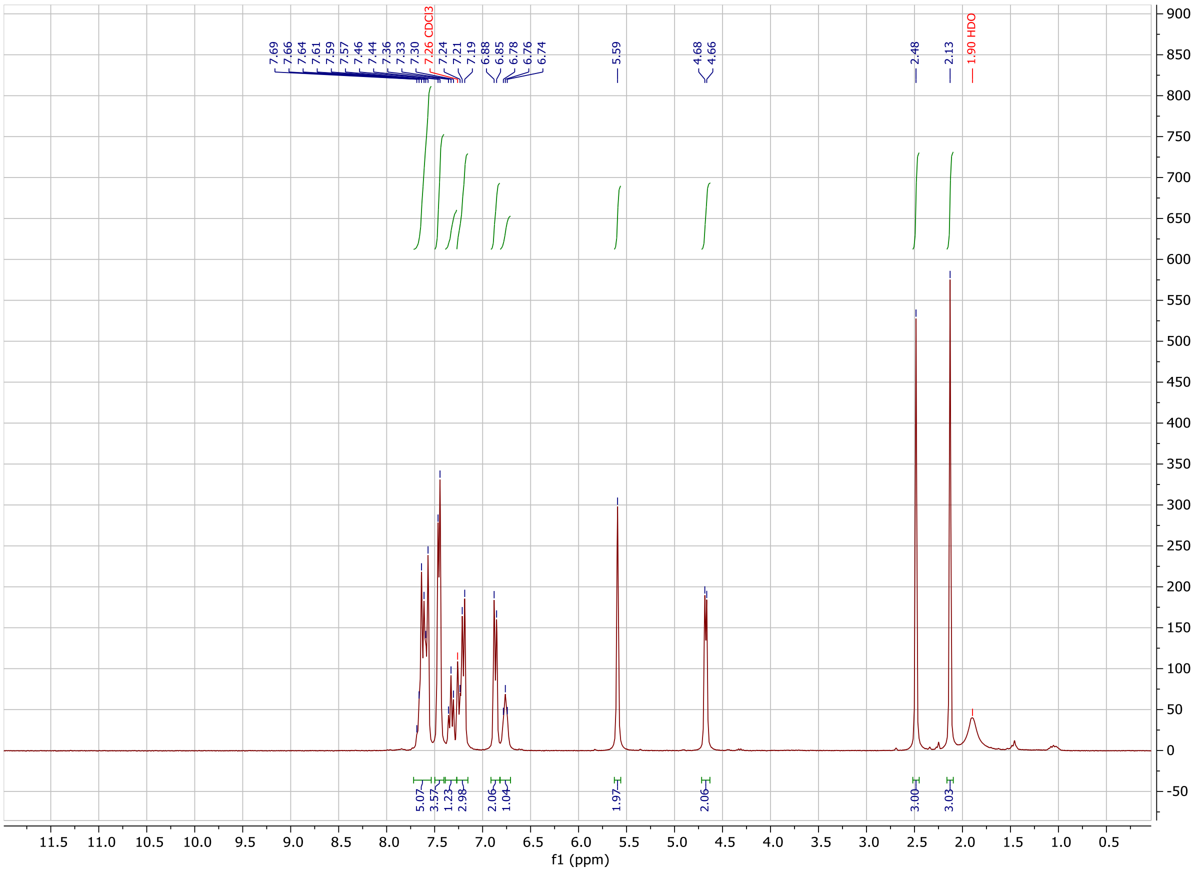

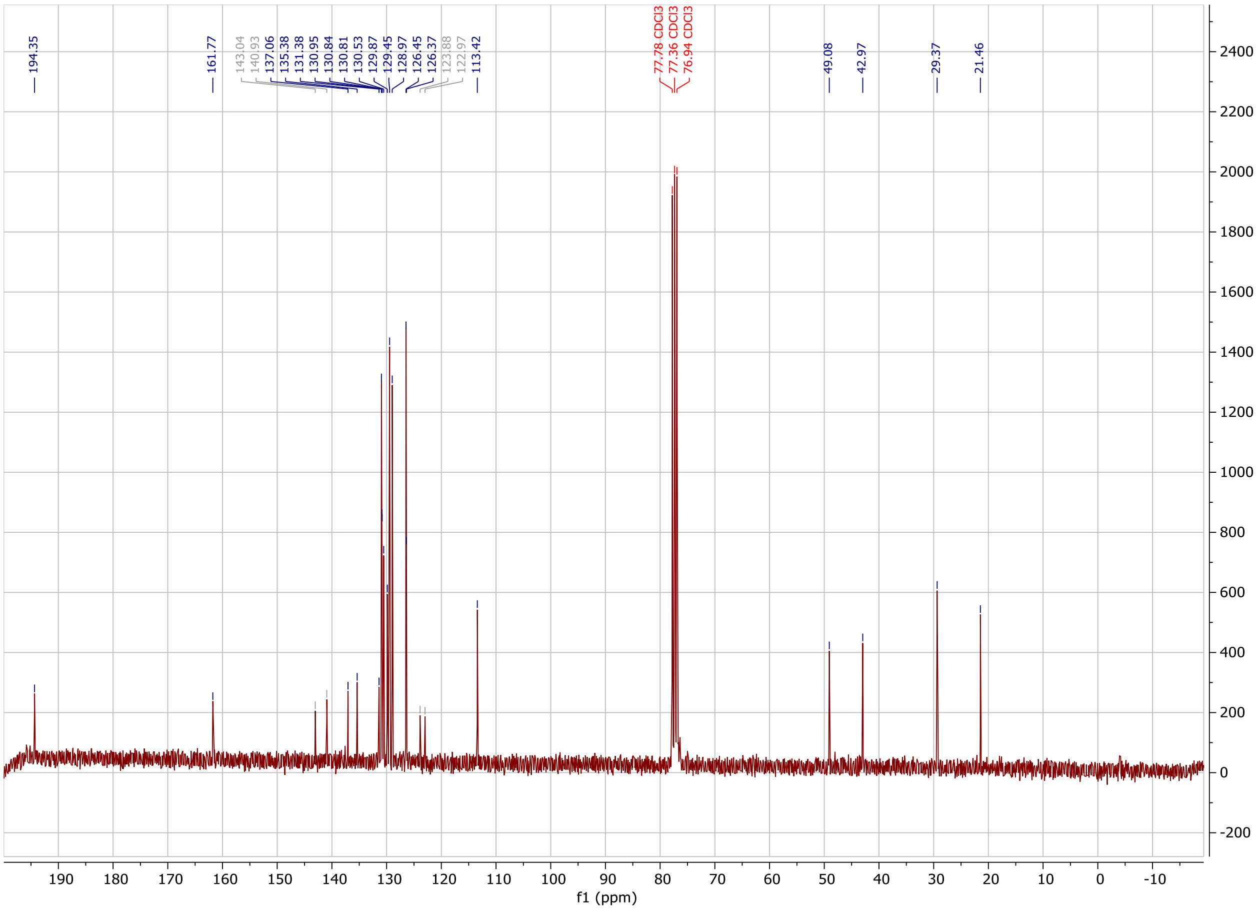

^{1}H NMR (300 MHz, $CDCl_3$) and ^{13}C NMR (75 MHz, $CDCl_3$) of compound **9g**

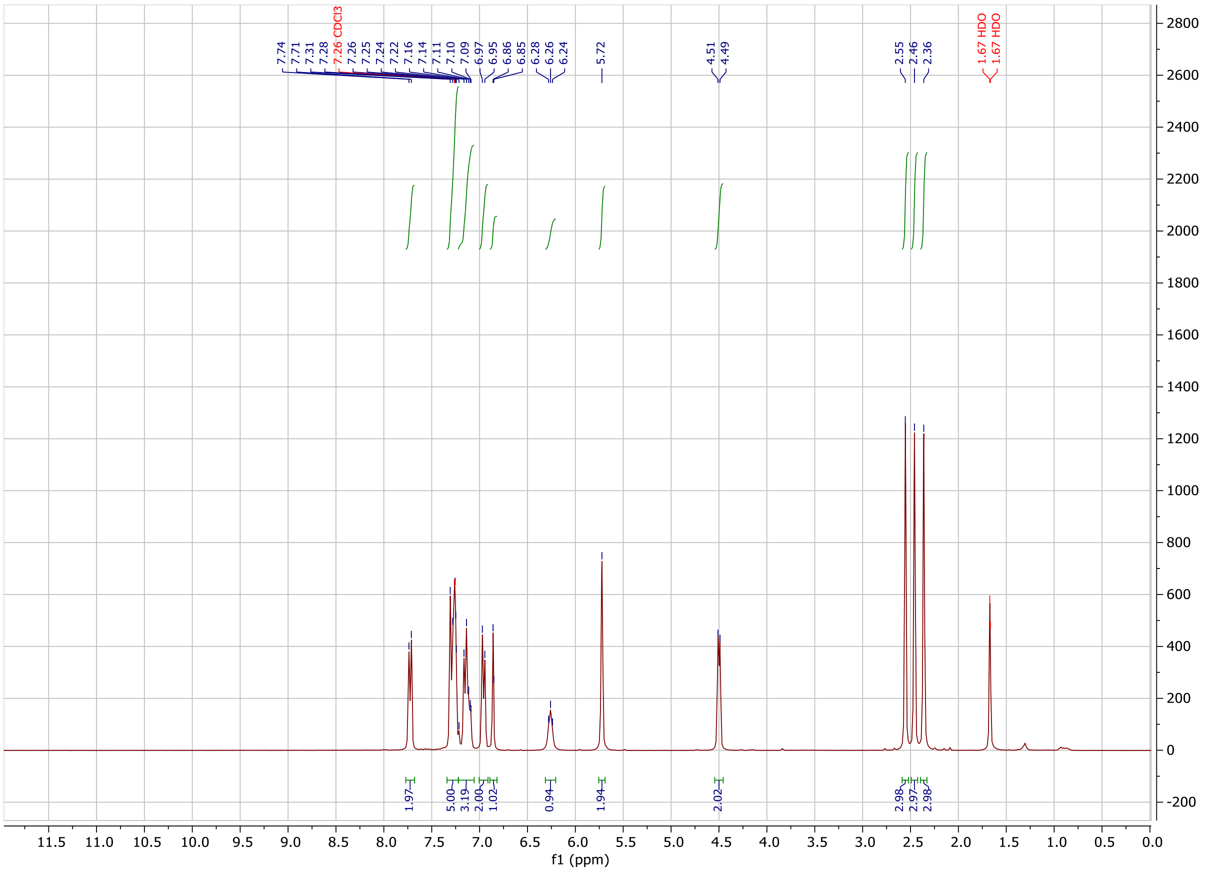

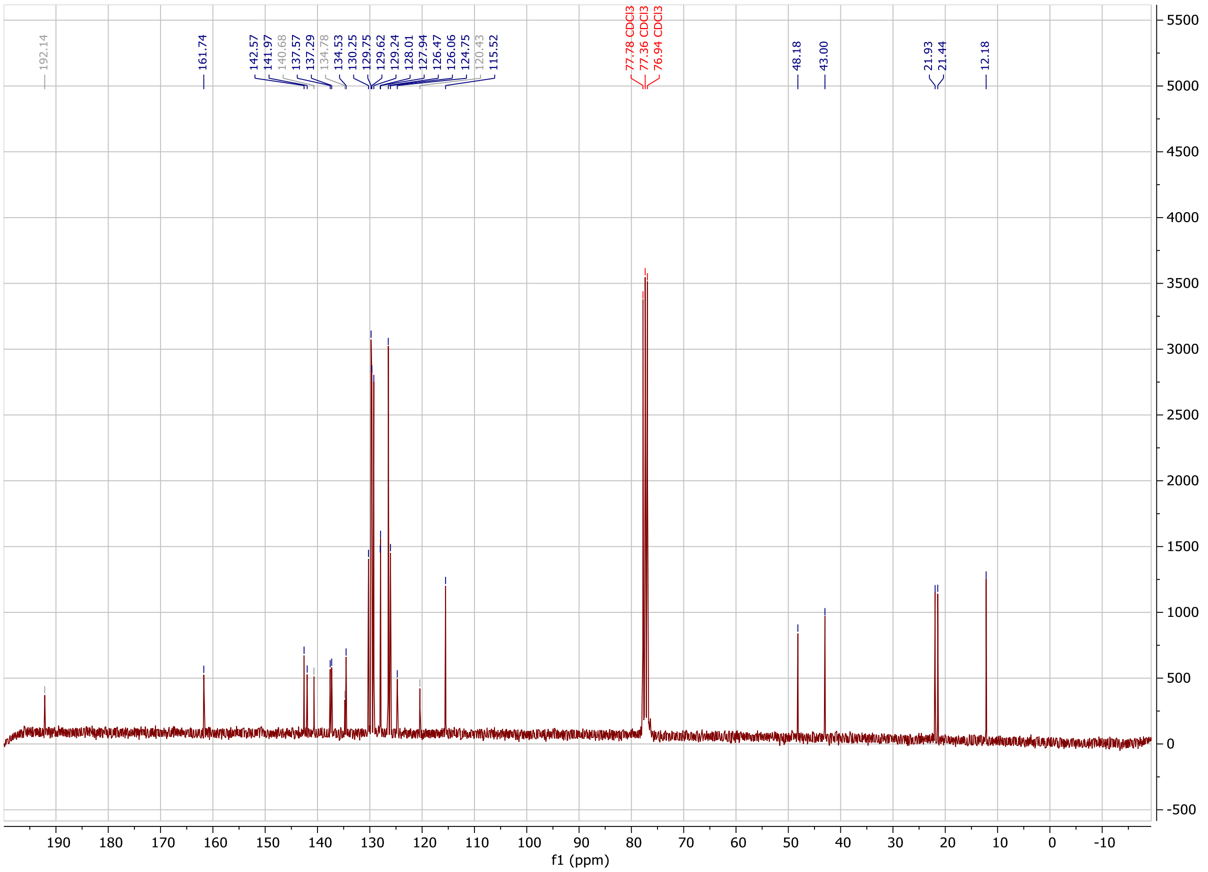

^{1}H NMR (300 MHz, $CDCl_3$) and ^{13}C NMR (75 MHz, $CDCl_3$) of compound **9h**

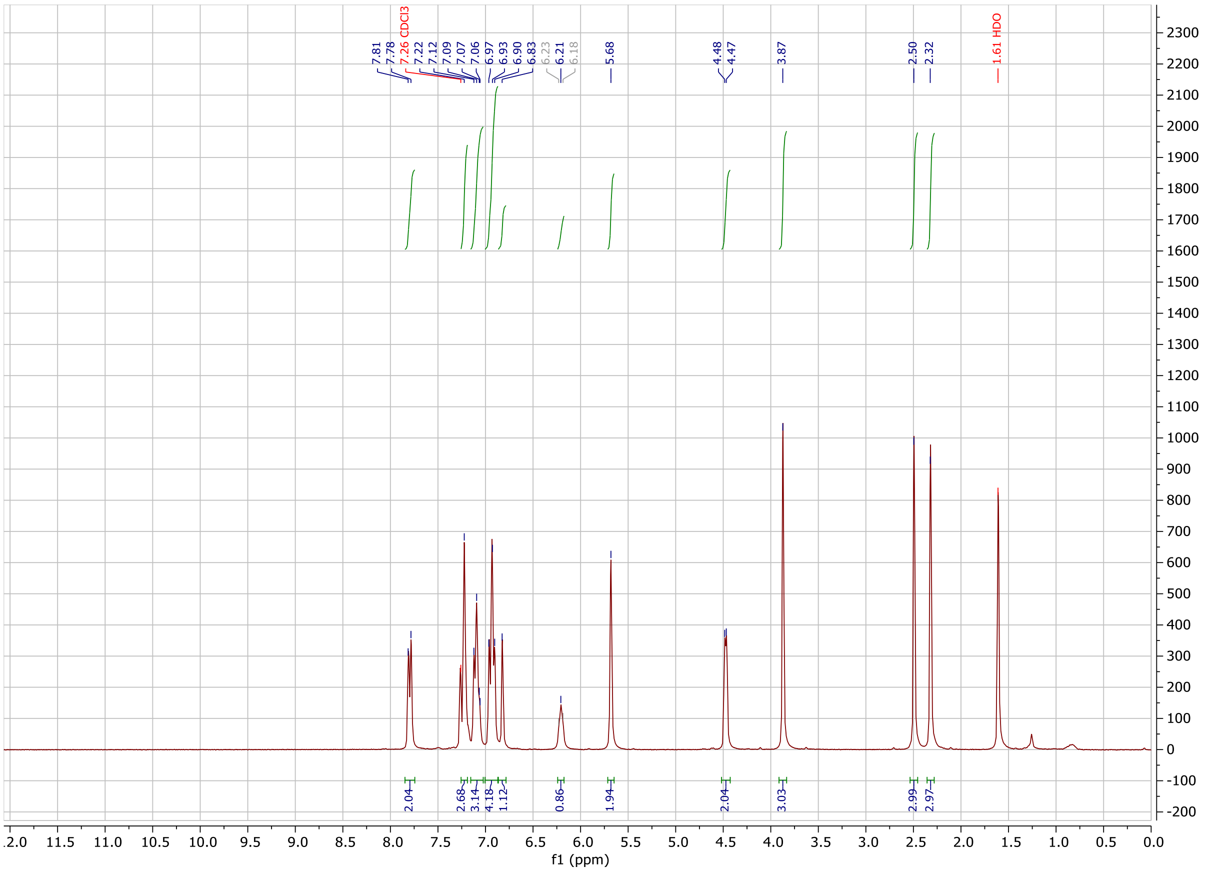

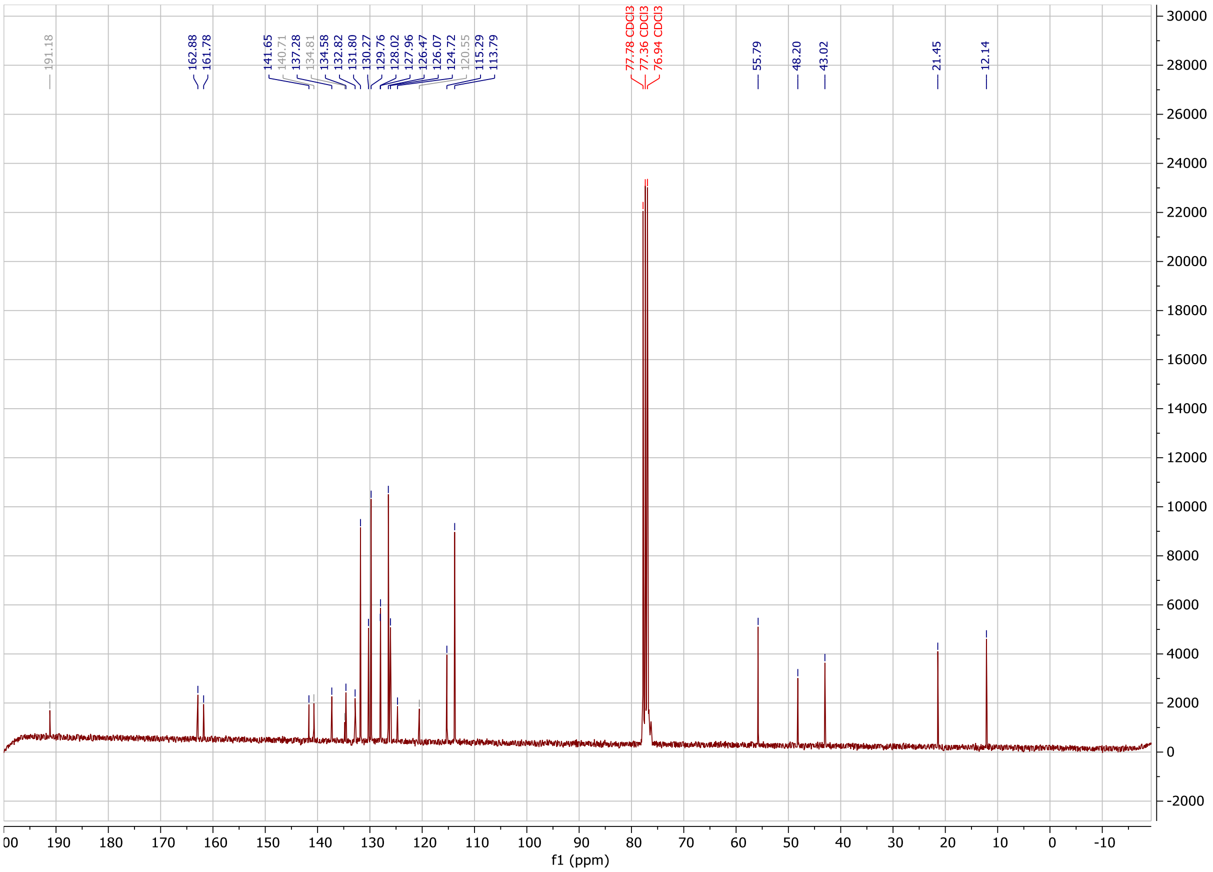

^{1}H NMR (300 MHz, $CDCl_3$) and ^{13}C NMR (75 MHz, $CDCl_3$) of compound **9i**

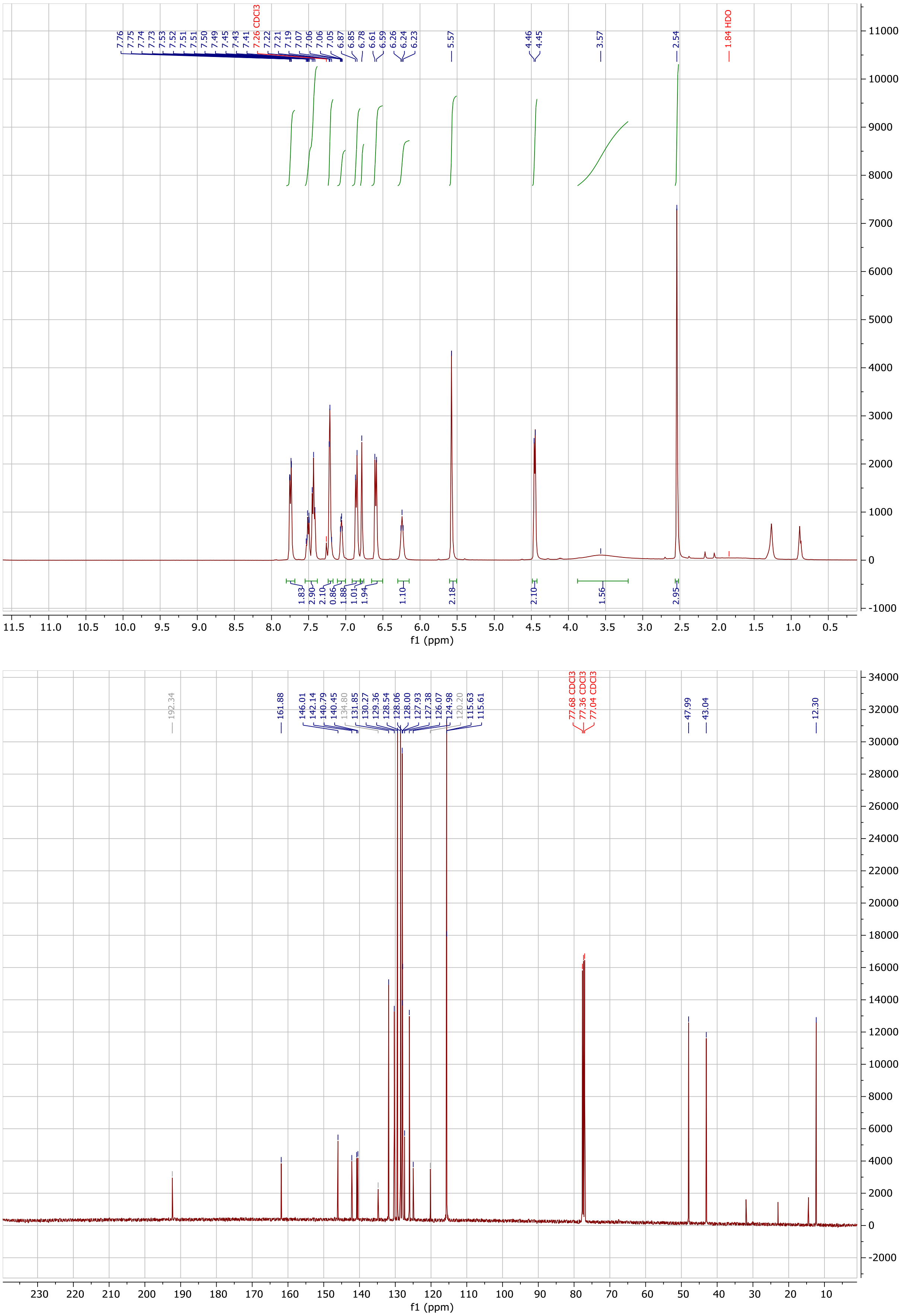

^{1}H NMR (400 MHz, $CDCl_3$) and ^{13}C NMR (101 MHz, $CDCl_3$) of compound **10a**

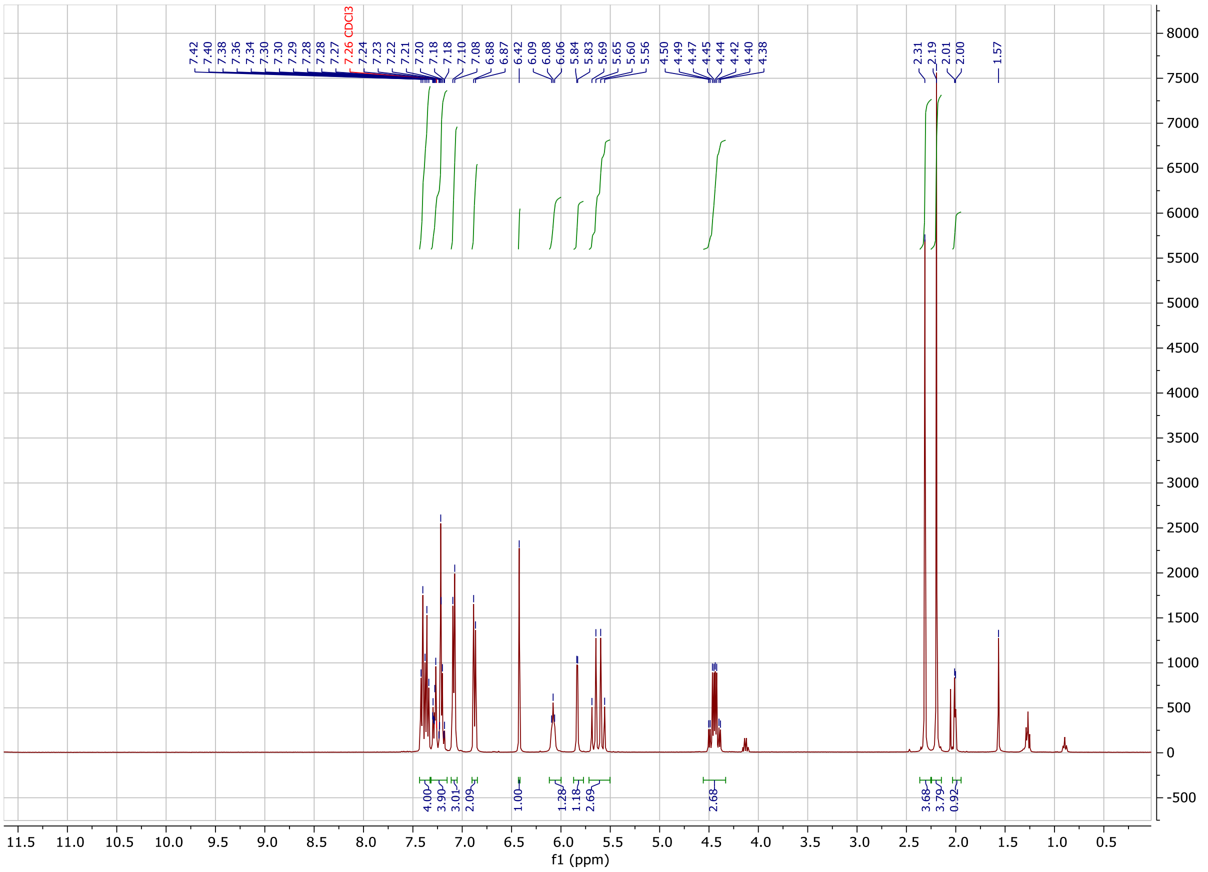

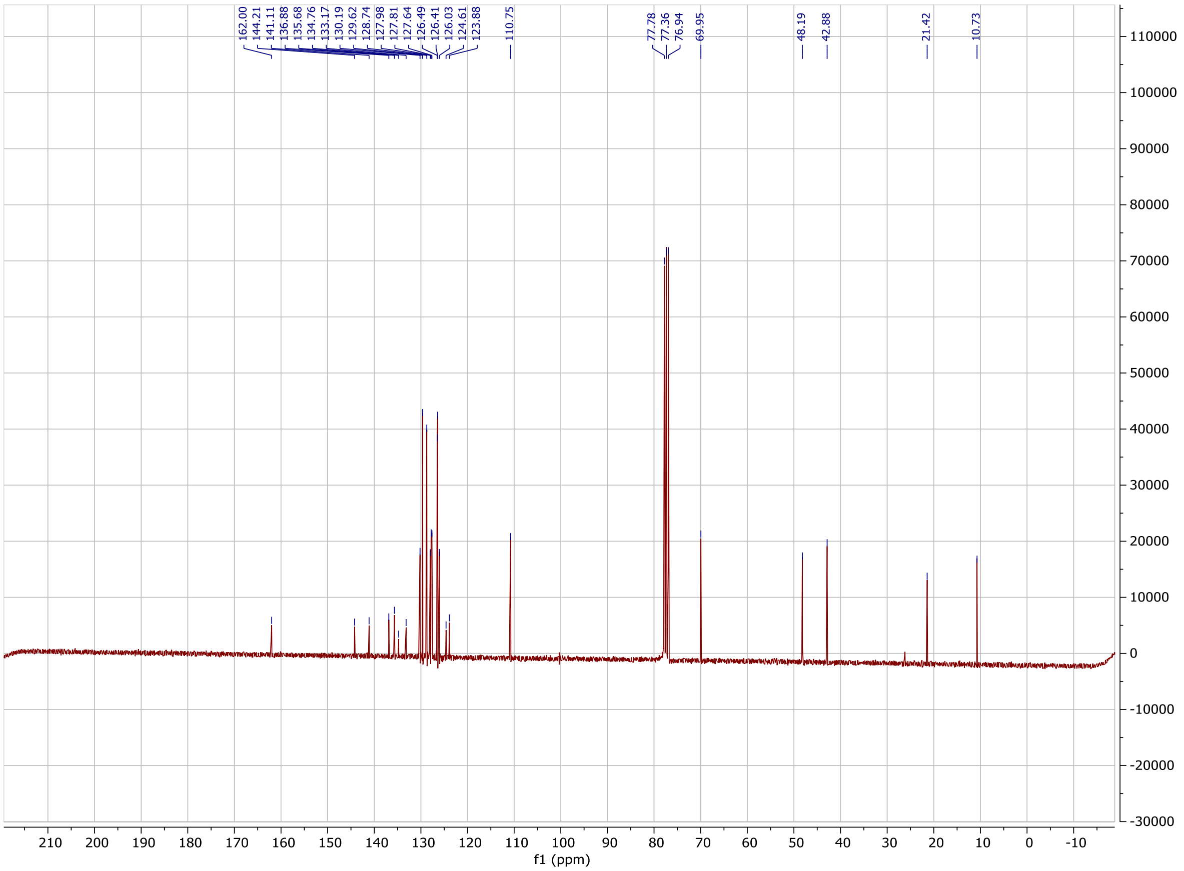

^{1}H NMR (400 MHz, $CDCl_3$) and ^{13}C NMR (101 MHz, $CDCl_3$) of compound ±**10b**
